# Sirtuin-1 in Hypertension: Linking Oxidative Stress and Mitochondrial Dysfunction to Vascular Aging and Therapeutic Potential

**DOI:** 10.3390/antiox15101353

**Published:** 2026-10-20

**Authors:** Sandra Budziak, Monika Kloza, Przemysław Baranowski, Oskar Antoniak, Aniqa Saiyara, Marta Żarczyńska-Buchowiecka, Marcin Hellmann, Barbara Kutryb-Zając, Marta Baranowska-Kuczko

**Affiliations:** 1 Department of Clinical Pharmacy, Medical University of Białystok, Mickiewicz Str. 2A, 15-089 Białystok, Poland; sandra.budziak@umb.edu.pl (S.B.); 41632@student.umb.edu.pl (O.A.); 2 Department of Experimental Physiology and Pathophysiology, Medical University of Białystok, Mickiewicz Str. 2A, 15-089 Białystok, Poland; monika.kloza@umb.edu.pl; 3 Department of Drug Addiction Pharmacology, Maj Institute of Pharmacology, Polish Academy of Sciences, Smętna Str. 12, 31-343 Kraków, Poland; aniqa.saiyara@if-pan.edu.pl; 4 Department of Cardiac Diagnostics, Medical University of Gdansk, Mariana Smoluchowskiego Str. 17, 80-214 Gdańsk, Poland; marta.zarczynska-buchowiecka@gumed.edu.pl (M.Ż.-B.);; 5 Department of Biochemistry, Medical University of Gdansk, Dębinki Str. 1, 80-211 Gdańsk, Poland; barbara.kutryb-zajac@gumed.edu.pl; 6 Centre of Experimental Cardiooncology, Medical University of Gdansk, Dębinki Str. 1, 80-211 Gdańsk, Poland

**Keywords:** sirtuin 1, hypertension, NAD^+^ metabolism, vascular homeostasis, oxidative stress, mitochondrial dysfunction, vascular aging

## Abstract

Hypertension is a multifactorial cardiovascular disorder characterized by oxidative stress, endothelial dysfunction, chronic inflammation, mitochondrial impairment, and accelerated vascular aging, all of which contribute to hypertension-mediated target organ damage. Among the molecular pathways involved in these processes, sirtuin-1 (SIRT1), a nicotinamide adenine dinucleotide (NAD^+^)-dependent deacetylase, has emerged as a potential regulator of vascular and metabolic homeostasis. This review summarizes current evidence on the role of SIRT1 in the pathophysiology of hypertension, with particular emphasis on its interactions with oxidative stress, mitochondrial function, endothelial integrity, inflammation, fibrosis, and cellular senescence. Experimental studies suggest that SIRT1 modulation can improve endothelial function and mitochondrial homeostasis, attenuate oxidative stress, inflammation, and vascular remodeling, and limit organ damage in several models. Effects on blood pressure are less consistent and appear to depend on the model and intervention used. Although clinical evidence remains limited, altered SIRT1 expression or activity has been reported in association with hypertension and preeclampsia, while preliminary studies suggest that interventions proposed to activate SIRT1 may improve vascular function and inflammatory status. However, changes in SIRT1 expression might not necessarily reflect changes in its enzymatic activity, and the effects of pharmacological interventions cannot always be attributed specifically to SIRT1. Overall, SIRT1 represents a potential therapeutic target whose value as a biomarker remains uncertain; however, well-designed clinical studies are required to establish its prognostic value and determine whether SIRT1 modulation can improve long-term cardiovascular outcomes.

## 1. Introduction

**Figure 1:**
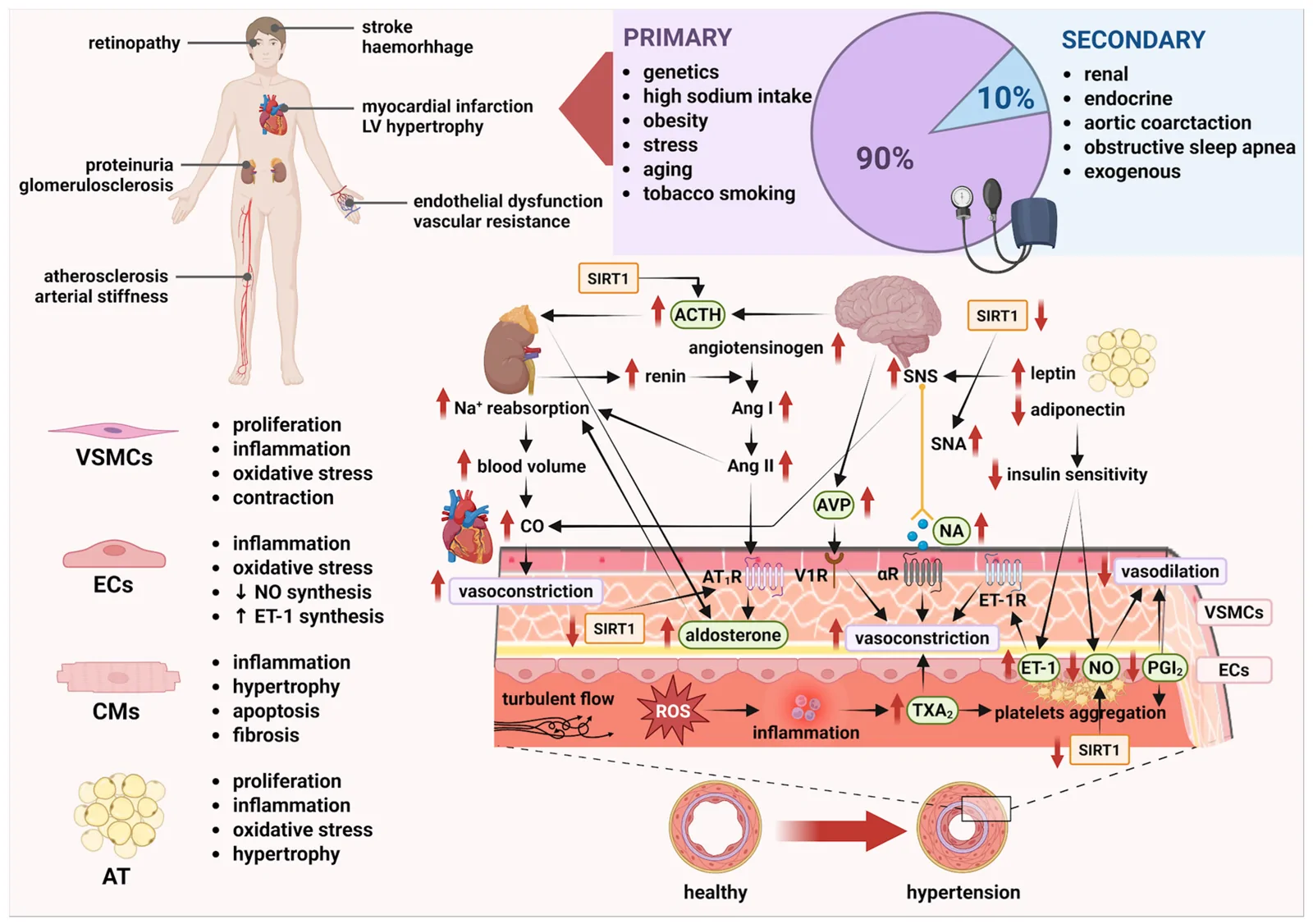
Overview of the causes, pathophysiology, cellular alterations, and target-organ damage associated with hypertension. SIRT1 labels indicate selected sites where SIRT1 may be involved; the remaining elements provide context and do not imply direct regulation by SIRT1. Abbreviations: ACTH—adrenocorticotropic hormone, αR—alpha adrenergic receptor, Ang—angiotensin, AT—adipose tissue, AT1R—angiotensin II receptor type 1, AVP—arginine vasopressin, CMs—cardiomyocytes, CO—carbon monoxide, ECs—endothelial cells, ET-1—endothelin 1, ET-1R—endothelin 1 receptor, LV—left ventricle, NA—noradrenaline, NO—nitric oxide, PGI2—prostaglandin I2/prostacyclin, ROS—reactive oxygen species, SIRT1—sirtuin 1, SNA—sympathetic nerve activity, SNS—sympathetic nervous system, TXA2—thromboxane A2, V1R—vasopressin receptor 1, VSMCs—vascular smooth muscle cells. Created in BioRender.com.

Hypertension is one of the most prevalent diseases worldwide, affecting almost 1.5 billion people [1]. It is diagnosed when resting systolic blood pressure (SBP) is equal to or above 140 mmHg and/or resting diastolic blood pressure (DBP) is equal or above 90 mmHg, and it can lead to several diseases, especially when untreated or treated inadequately [2]. It is an important risk factor for cardiovascular diseases (CVD) and accounts for 20% of deaths worldwide [3]. Most patients develop primary hypertension, while only approximately 10% are diagnosed with secondary hypertension [2]. Essential hypertension risk factors include modifiable lifestyle factors such as low adherence to the Dietary Approaches to Stop Hypertension diet and high sodium intake, excessive alcohol consumption, smoking, obesity and physical inactivity [4]. Moreover, lack of disease awareness and resistance to lifestyle changes can contribute to the development of hypertension in individuals with elevated blood pressure [2]. Hypertension management includes lifestyle modifications and pharmacological therapy. When starting treatment, laboratory tests should be performed to screen for secondary hypertension and possible comorbidities, and cardiovascular (CV) risk should be assessed [5]. The 2024 European Society of Cardiology document emphasizes hypertension as a continuous exposure scale and therefore guides to treat not only individuals meeting the criteria of hypertension but also for patients with elevated blood pressure (BP) defined as an office SBP 120–139 mmHg or DBP of 70–89 mmHg, based on CV risk assessment [2]. To date, it is well established that both modifiable and non-modifiable risk factors contribute to the development and progression of elevated BP and hypertension. Non-modifiable factors include ageing and genetic predisposition, whereas modifiable factors include physical inactivity, excess body weight, dietary factors, high sodium intake, alcohol consumption, and smoking. Non-pharmacological interventions addressing these modifiable factors, including increased physical activity, weight reduction, dietary optimization with enhanced potassium intake and reduced sodium consumption, decreased alcohol intake, and smoking cessation, translate into clinically meaningful benefits in terms of BP reduction and overall CV risk reduction [4]. Pharmacological therapy with proven clinical outcome benefits in the target population includes angiotensin converting enzyme (ACE) inhibitors/angiotensin II (Ang II) receptor blockers, dihydropyridine calcium channel blockers, diuretics (thiazide and thiazide-like), and β-blockers [2]. Beyond established second-line drugs such as spironolactone, newer agents including sacubitril/valsartan [6] and sodium–glucose cotransporter 2 (SGLT2) inhibitors [7] have shown promising effects in the treatment of patients with hypertension, though supportive evidence from CV outcomes trials is still awaited [2]. Despite the availability of safe and cost-effective therapies, 2/3 of patients with hypertension are undiagnosed, untreated, or under treated [3]. High BP increases the risk of many CVD, including coronary heart disease and heart failure (HF), and is also associated with complications such as kidney disease and stroke. Furthermore, hypertension is related to an increased risk of dementia and cognitive impairment [8]. Taken together, these findings highlight the importance of effective hypertension prevention and treatment in reducing the risk of a wide range of diseases. The pathophysiology involves complex interactions between environmental and behavioral factors, genetic predisposition, hormonal networks, and multiple organ systems including the renal, CV, as well as central nervous systems and remains a leading global risk factor for CV morbidity and mortality, with endothelial dysfunction recognized as a central mechanism in its pathogenesis [2,9,10]. Dysregulation in these mechanisms can contribute to hypertension-mediated organ damage (HMOD) and unfavorable CV outcomes [2] [Figure 1].

The development of hypertension is closely related to functional and structural changes in the vasculature. High BP results in the endothelial dysfunction, vascular stiffness, remodeling of resistance arteries and heart and vascular hypertrophy, all of which contribute to increased peripheral vascular resistance and sustained elevation of BP [11,12,13,14]. Impaired bioavailability of nitric oxide (NO), enhanced vasoconstrictor signaling, and abnormal regulation of vascular tone play a crucial role in the progression of hypertension [11,15]. At the molecular level, several signaling pathways are involved in the regulation of vascular function and BP, particularly NO, renin–angiotensin–aldosterone (RAA) system, nicotinamide adenine dinucleotide phosphate (NADPH) oxidase (NOX) and tumor necrosis factor-α (TNFα) signalling [16]. Oxidative stress, chronic low-grade inflammation, and disturbances in metabolic regulation are considered key mechanisms contributing to the development of hypertension [17,18]. Excessive production of reactive oxygen species (ROS) leads to reduced NO bioavailability and promotes endothelial dysfunction, while inflammatory signaling and hormonal imbalance further enhance vascular remodeling and impair normal vascular reactivity [1,17,19,20]. In recent years, increasing attention has been focused on the role of epigenetic regulation and metabolic sensors in CVD, including hypertension [21,22]. In the context of ongoing efforts in experimental cardiology to identify optimal therapeutic strategies and given the important role of the endothelium in the pathogenesis of hypertension, the sirtuin 1 (SIRT1) molecule has recently attracted particular attention. It has emerged as an important regulator of cellular metabolism, oxidative stress response, inflammation, and vascular homeostasis. SIRT1 is widely expressed in CV tissues, including endothelial cells (ECs), vascular smooth muscle cells (VSMCs), cardiomyocytes, and renal tissue, where it modulates multiple signaling pathways essential for maintaining normal vascular function and BP regulation [23,24]. Experimental and clinical evidence suggests that altered SIRT1 activity may contribute to the development of hypertension through its effects on endothelial function, vascular remodeling, oxidative stress, inflammatory response, and neuronal regulation [17,25,26,27,28,29,30].

Moreover, pharmacological activation of SIRT1 has been shown to exert protective effects in various models of CVD, indicating that this molecule may represent a promising therapeutic target [31].

This review summarizes current knowledge on the role of SIRT1 in the pathophysiology of hypertension, with particular emphasis on its involvement in oxidative stress, inflammation, mitochondrial function, and cell survival and aging, as well as the potential therapeutic implications of targeting SIRT1 in the treatment of hypertension.

## 2. Materials and Methods

To identify the most relevant articles on the role of SIRT1 in hypertension, PubMed database was searched. This review was designed as a structured narrative review focusing on the role of SIRT1 in hypertension, with particular emphasis on oxidative stress, mitochondrial dysfunction, endothelial dysfunction, inflammation, vascular remodeling, vascular ageing, and cellular senescence. Publication dates were restricted to the last 5 years to include the most recent studies with novel findings; however, for human studies, the publication date range was extended to include all available articles. The searches were conducted on 30 January 2026.

The term “SIRT1” was used as the primary keyword, and the search was refined by adding the secondary terms “systemic” and “hypertension” with the Boolean operator AND, which yielded 169 results in total. To broaden the search and reduce the risk of omitting relevant studies, additional synonyms and terms related to hypertension and SIRT1-associated mechanisms were subsequently considered, including “sirtuin 1”, “silent information regulator 1”, “blood pressure”, “elevated blood pressure”, “vascular hypertension”, “endothelial dysfunction”, “oxidative stress”, “mitochondrial dysfunction”, “inflammation”, “vascular remodeling”, “vascular ageing”, and “cellular senescence”. Boolean operators AND and OR were used to combine the search terms. Then, we extended date range and added the terms “patients” or “human”, or combined the word “SIRT1” with “preeclampsia” in order to find more articles on human subjects, which yielded 318 more results. We also searched the ClinicalTrials.gov database to identify studies investigating SIRT1-targeting or SIRT1-related interventions in hypertension. A detailed description of the complete search strategy, including all search strings and queries applied for each database, is provided in Supplementary Table S1.

Studies were eligible for inclusion in the primary evidence synthesis if they (1) were original research articles, (2) were published in English, (3) investigated SIRT1 in the context of hypertension or hypertension-related cardiovascular mechanisms, and (4) provided experimental or clinical data relevant to the role, regulation, or effects of SIRT1. Studies were excluded if they (1) were not directly related to SIRT1 and hypertension or hypertension-related cardiovascular mechanisms, (2) focused exclusively on diseases unrelated to hypertension, (3) were not original research articles, (4) were published in languages other than English, or (5) did not provide relevant information regarding SIRT1 expression, activity, regulation, or functional effects in the context of hypertension.

Only full-text original articles were included. First, the titles were analysed, then the abstracts and full texts of articles, and not retrieved, duplicated or ineligible papers were removed. Subsequently, we excluded non-relevant records based on titles and abstracts. These included studies in language other than English, referred to SIRT1 role in diseases other than hypertension, or did not provide information about SIRT1 effects in hypertension.

The records identified through the database searches were screened in a stepwise manner. Following removal of duplicate records, titles were screened for relevance, and potentially eligible articles were subsequently assessed in full text. 2604 duplicate records were removed, 3502 records were screened based on title, 115 full-text articles were assessed for eligibility. At the abstract and full-text stage, 64 studies were excluded for the following reasons: (1) were not directly related to SIRT1 and hypertension or hypertension-related cardiovascular mechanisms; (2) focused exclusively on diseases unrelated to hypertension; (3) did not provide relevant information regarding SIRT1 expression, activity, regulation, or functional effects in the context of hypertension.

Finally, 51 publications were included in this review and are demonstrated in Tables in the subsections “Effects in Rodents” and “Effects in Humans”. These 51 publications represent the primary studies included in the focused evidence synthesis and should be distinguished from additional references cited elsewhere in the manuscript. Further background details were derived from additional reviews and original articles listed in the references. Earlier original studies and review articles were additionally considered where necessary to provide foundational information on SIRT1 biology, established molecular mechanisms, historical context, and relevant physiological or clinical concepts. These background references were not considered part of the 51 primary publications included in the focused evidence synthesis.

Because this manuscript is a structured narrative review rather than a formal systematic review, the literature was synthesized narratively, with emphasis on the biological context, consistency of findings across experimental models, and translational relevance of the available evidence. Particular attention was given to differences between genetic and pharmacological SIRT1 modulation, acute and chronic SIRT1 activation, SIRT1 expression and enzymatic activity, the influence of NAD^+^ availability, and potential differences between experimental models and cell types. For the figure preparation, the software used was BioRender.com.

## 3. Sirtuin 1

The mammalian sirtuin (SIRT) family comprises seven distinct NAD^+^-dependent deacetylases (SIRT1–SIRT7) that differ in their subcellular localization, enzymatic activities, and primary tissue targets. SIRT1 and SIRT2 predominantly reside in the nucleus and cytoplasm, whereas SIRT3, SIRT4, and SIRT5 are localized within the mitochondrial matrix, and SIRT1 and SIRT7 are nuclear proteins [32]. Deacetylation is the main catalytic activity of SIRT1, SIRT2, SIRT3, SIRT4, SIRT6 and SIRT7. Beyond classic deacetylation, these isoforms possess specialized post-translational modification activities: SIRT3 mediates protein decrotonylation; SIRT4 acts also via adenosine diphosphate (ADP)-ribosylation; SIRT5 exhibits distinct desuccinylation, demalonylation, and deglutarylation activities; and SIRT6 displays ADP-ribosylation and deacylation capacity. Among these isoforms, SIRT1 remains the most extensively characterized in CV physiology due to its broad expression in endothelial and vascular smooth muscle cells, its central position downstream of calorie restriction and NAD^+^ metabolism, and its diverse target spectrum—deacetylating key regulatory factors such as endothelial nitric oxide synthase (eNOS), nuclear factor kappa-light-chain-enhancer of activated B cells (NF-κB), peroxisome proliferator-activated receptor-γ coactivator 1-α (PGC-1α), and forkhead box protein O (FOXO) family proteins [31,33]. Meanwhile, SIRT7, the latest discovered SIRT family protein, has been relatively less studied [32].

**Figure 2:**
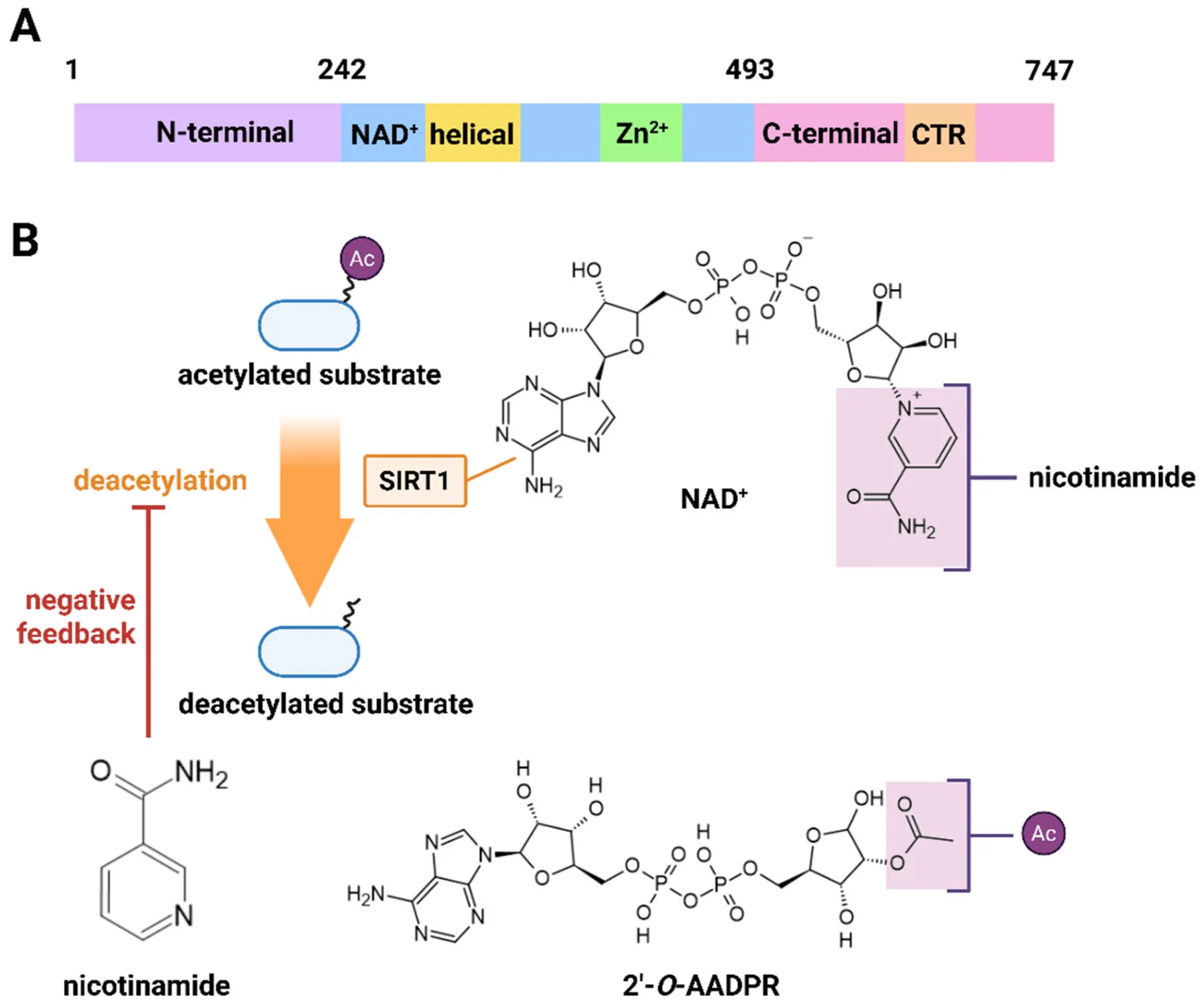
(**A**) Molecular structure of SIRT1. N-terminal segment (residues 1 to 242) is shown in purple, NAD^+^-binding region (residues 244 to 268, 325 to 361 and 419 to 493) is shown in blue, helical module (residues 269 to 324) is shown in yellow, Zn^2+^-binding region (residues 362 to 418) is shown in green, C-terminal region (residues 494 to 640 and 666 to 747) is shown in pink, and C-terminal regulatory region (residues 641 to 665) is shown in orange. (**B**) Mechanism of SIRT1 deacetylation process. Created in BioRender.com.

SIRT1 contains 747 amino acid residues and consists of four domains: NAD^+^ binding, catalytic, NH2-terminal and COOH terminal [Figure 2] [34]. The catalytic efficiency of the SIRT1 catalytic domain can be enhanced by the N-terminal and C-terminal segments. N-terminal domain increases catalytic constant (kcat), while the C-terminal region lowers the Michaelis constant (Km) for NAD^+^ [35], increasing the affinity. The catalytic domain of SIRT1 consists of 277 residues, organized into a C-terminal regulatory segment (CTR) and two other subdomains. These subdomains include a larger, NAD^+^-binding domain, and a smaller domain created by two formations within the NAD^+^-binding region, namely a helical module and a Zn^2+^-binding module [36].

The catalytic core can exist in two states: “open” and “closed”. When there is no cofactor or substrate present, the smaller domain undergoes a ≈25° rotation, creating a groove between the subdomains that exposes the unchanging hydrophobic interior and the NAD^+^-binding site. During the SIRT1 deacetylation process, the acetyl group of the substrate is moved onto the ribose part of NAD^+^, resulting in the formation of nicotinamide (NAM), 2′-O-acetyl-ADP-ribose (2′-O-AADPR) and deacetylated substrate [37]. The activity of SIRT1 can be inhibited by NAM via a negative feedback mechanism [38].

SIRT1 activity is controlled by CTR. It consists of 25 residues and includes a sizable N-terminal segment and a shorter C-terminal extension at the end, both engaging in separate interactions with the catalytic domain. The N-terminal segment creates a β hairpin structure that enhances the central parallel β sheet within the CTR’s Rossmann fold domain by incorporating two further strands, while the C-terminal extension engages in additional binding with the helical module visible only in one crystallized state [37].

SIRT1 is widely expressed throughout the body, including the brain, lungs, muscles, liver, spleen, and vasculature [39]. It is predominantly localized in the nucleus but shuttles between the nucleus and cytoplasm, playing a key role in maintaining cellular homeostasis through regulation of metabolism, deacetylation of histones, and modulation of downstream targets to exert cytoprotective effects [40,41]. Numerous studies have highlighted SIRT1’s central role in metabolic regulation. Both exercise and caloric restriction increase the NAD^+^/NADH ratio, which directly activates SIRT1. Activated SIRT1 deacetylates PGC-1α and enhances fatty acid oxidation via peroxisome proliferator-activated receptor α (PPARα), promoting metabolic fitness [42,43].

SIRT1 can be activated by natural compounds found in foods as well as by synthetic molecules. Natural activators, including resveratrol (REV), fisetin, and berberine, are more frequently studied [44,45] and new modulators continue to be discovered [46]. Synthetic activators, such as SRT1720 and SRT2104, exhibit potency thousands of times greater than natural compounds [47,48]. However, many currently studied modulators are not fully selective and may also influence sirtuin 2 (SIRT2) and sirtuin 3 (SIRT3), which are abundant in metabolically active tissues like the heart, brain, and skeletal muscles [48,49]. SIRT1 is primarily activated by increased NAD^+^ levels, although some compounds—including SRT1720, SRT2104, REV, and alpinumisoflavone—can allosterically activate the enzyme [50,51].

Most SIRT1 modulators share similar protective effects, including antioxidative, anti-inflammatory, antiproliferative, vasodilatory, and hypotensive actions (Table 1).

SIRT1 modulation was predominantly associated with antioxidative, anti-inflammatory, and antiproliferative effects, whereas effects on blood pressure, vascular tone, migration, and apoptosis were less consistent and included both protective and opposite responses (Table 1).

Increased NAD^+^ availability was reported for several SIRT1 modulators, including REV [52], amifostine [53], quercetin [54], and berberine [55]. Antioxidative effects were among the most consistently reported outcomes of SIRT1 modulation [54,56,57,58,59,60,61,62,63,64,65,66,67,68,69]. Anti-inflammatory effects were frequently reported among SIRT1 modulators, although opposite or context-dependent effects were also observed for some compounds [70,71,72,73,74,75,76,77,78,79,80,81,82,83,84,85]. Antiproliferative effects were reported for several SIRT1 modulators, including REV [86], quercetin [87], berberine [88], ferulic acid [89], SRT1720 [90], SRT2104 [79], Jagged 2 inhibition [65], and Valine—Histidine—Valine—Valine peptide (VH4) [83]. In contrast, amifostine [91], fisetin [92], and Klotho [93] showed no antiproliferative effect, while 15-HETE exerted a pro-proliferative effect [94]. Hypotensive and vasodilatory effects were reported for several modulators, although these effects were less consistently documented than antioxidative or anti-inflammatory actions [78,79,86,87,92,94,95,96,97,98]. The effects of SIRT1 modulation on apoptosis were heterogeneous, with both pro- and anti-apoptotic responses reported [58,65,74,75,79,83,86,87,98,99,100,101]. SIRT1 modulation occurred through several distinct mechanisms, including direct binding/activation [50,51], increased catalytic activity [102], increased SIRT1 expression [103], and indirect modulation through the NAD^+^/nicotinamide phosphoribosyltransferase (NAMPT)/AMP-activated protein kinase (AMPK) axis [90,104,105,106,107].

Hemodynamic, vasodilatory, and apoptotic effects are less uniform and appear to depend on the specific modulator and experimental context.

Overall, the available evidence suggests that SIRT1 modulation is associated with a broad spectrum of protective effects, particularly against oxidative stress, inflammation, and abnormal vascular cell proliferation, while the underlying mechanisms and functional outcomes remain modulator- and context-dependent.

**Table 1:** Modulators of SIRT1 and their functions.

Modulator	Mechanism		Effect (Presence, Ref.)						
	↑ NAD^+^	Other	Antioxidative	Anti-Inflammatory	Antiproliferative	Hypotensive	Vasodilatory	Antimigratory	Apoptotic
resveratrol	+ [52]	↑ direct binding to SIRT1 [51]	+ [56]	+ [70]	+ [86]	+ [86]	+ [108]	n/d	n/d
amifostine	+ [53]	n/d	+ [57]	− [71]	+ [91]	+ [95]	− [109]	n/d	n/d
quercetin	+ [54]	n/d	+ [54]	+ [72]	+ [87]	+ [96]	+ [110]	+ [87]	+ [87]
berberine	+ [55]	activation of AMPK [106]	+ [58]	+ [73]	+ [88]	+ [97]	+ [97]	n/d	− (anti-) [58]
NAMPT	+ [104]	n/d	+ [111]	− (proinflammatory) [112]	− (proliferative) [113]	+ [114]	− (vasoconstrictor) [115]	− (migratory) [113]	n/d
fisetin	n/d	↑ catalytic activity of SIRT1 [102]	+ [59,60]	+ [59]	+ [74]	− [92]	+ [92]	+ [59]	+ [74]
AMPK	+ [116]	↑ NAMPT [105]	+ [117]	+ [118]	+ [119]	+ [120]	+ [121]	+ [122]	− (anti-) [123]
curcumin	n/d	activation of AMPK [107]	− (pro-) [75] + [61]	+ [75,76]	−/+ [107]	+ [124]	+ [76,125]	n/d	+ [75]
ferulic acid	n/d	↑ SIRT1 mRNA [126]	+ [62]	+ [77]	+ [89]	+ [127]	+ [128]	n/d	n/d
SRT1720	n/d	deacetylation of SIRT1 substrates [49]	+ [63]	+ [78]	+ [90]	+ [86]	+ [78]	n/d	+ [86]
SRT2104	n/d	direct binding to SIRT1 (allosteric activation) [50]	+ [64]	+ [64]	+ [79]	+ [79]	− [129]	n/d	+ [79]
Stac-3	n/d	↑ NAMPT [90]	n/d	n/d	+ [90]	n/d	n/d	n/d	− (anti-) [99]
Jagged 2 inhibition	n/d	n/d	+ [65]	+ [80]	+ [65]	+ [65]	n/d	n/d	+ [65] − [100]
15-HETE	n/d	↑ transcription and translation of SIRT1 [103]	− (pro-) [81]	− (pro-) [82] + [66]	− [94] − (pro-) [130]	− (hypertensive) [98]	− (contractile) [94]	n/d	+ [98]
VH4	n/d	n/d	n/d	+ [67]	n/d	+ [83]	n/d	n/d	− (anti-) [83]
IF	n/d	n/d	n/d	+ [68]	n/d	+ [84]	n/d	n/d	− (anti-) [101]
Klotho	n/d	n/d	+ [69]	+ [85]	− [93]	n/d	n/d	n/d	n/d

Abbreviations: +, present; −, not present; n/d, not determined; 15-HETE, 15-Hydroxyeicosatetraenoic acid; AMPK, AMP-activated protein kinase; IF, Isoleucine-Phenylalanine dipeptide; NAD^+^, nicotinamide adenine dinucleotide; NAMPT, nicotinamide phosphoribosyltransferase; SIRT1, sirtuin 1; VH4, Valine—Histidine—Valine—Valine peptide.

Aging is strongly associated with a progressive decline in physiological functions and homeostatic mechanisms. SIRT1 is a well-established longevity-associated protein involved in the regulation of cellular aging, and its expression decreases with age, which may contribute to an increased susceptibility to age-related diseases [39]. Notably, SIRT1 indirectly inhibits mechanistic target of rapamycin (mTOR) signaling through modulation of AMPK activity, thereby promoting autophagy and delaying cellular senescence [131]. Conversely, SIRT1 itself can serve as a substrate for autophagic degradation, which may partly explain the age-related decline in its expression. This highlights a critical aspect of autophagy: although generally beneficial, excessive or non-selective degradation of key regulatory proteins such as SIRT1 may lead to unfavorable outcomes [132]. Cellular senescence is characterized by irreversible cell cycle arrest induced by telomere dysfunction, DNA damage, or oxidative stress [132,133]. Importantly, SIRT1 activation mitigates cell cycle arrest and delays cellular aging [125]. A recent study demonstrated that the active regulator of SIRT1 (AROS) antagonizes SIRT1 ubiquitination and prevents its degradation, thereby suppressing stress-induced premature senescence [133]. Moreover, arterial endothelial function is correlated with the bioavailability of NO, which decreases with aging and can be associated with altered SIRT1 function and therefore deactivated eNOS [134]. In our recent paper, we emphasized that REV has been documented to stimulate endothelial NO generation and inhibit oxidative stress, suggesting that antioxidant regimens can effectively counteract the adverse clinical effects of age-related microvascular endothelial dysfunction [135]. In primary vascular cells, activation of SIRT1 directly inhibits premature cellular senescence by reducing senescence-associated β-galactosidase (SA-β-gal) activity in aorta [136], downregulating tumor protein p53 (p53) in aorta [136] and human umbilical vein endothelial cells (HUVECs) [137] and cyclin-dependent kinase inhibitor 1A (p21) signaling pathways in aortic cells [136], and increasing NO bioavailability [138]. Furthermore, in VSMCs, SIRT1 activation suppresses Ang II-induced senescence and excessive cell proliferation [139] while downregulating NADPH oxidase components, such as NOX2 [137,140] and p47phox [141].

The pathogenesis of many diseases is associated with oxidative stress, which reduces intracellular NAD^+^ levels and consequently diminishes SIRT1 activity [142]. SIRT1 promotes antioxidant defense by targeting multiple substrates, including FOXO1, which enhances manganese superoxide dismutase (MnSOD) expression and stimulates SIRT1 transcription through a positive feedback loop [143,144,145]. Additionally, SIRT1-mediated deacetylation of PGC-1α and forkhead box O3 (FOXO3) increases the expression of antioxidant enzymes such as superoxide dismutase (SOD) and glutathione peroxidase (GSH), contributing to reduced ROS production [145,146]. Moreover, activation of the SIRT1/nuclear factor erythroid 2-related factor 2/heme oxygenase-1 (SIRT1/Nrf2/HO-1) signaling axis plays a pivotal role in alleviating oxidative stress and exhibiting cytoprotective effects [28,147].

An increasing amount of evidence links inflammation to the pathophysiology of numerous diseases, including CVD. In conditions characterized by chronic inflammation, tissues remain in a persistent pro-inflammatory phase without transition to an adaptive state, and dysregulation of the NAD^+^-sensing network leads to suppressed SIRT1 expression [148]. SIRT1 modulates key inflammatory regulators, including NF-κB, and attenuates the release of pro-inflammatory cytokines such as TNF-α, interleukin (IL)-1β, IL-6 and monocyte chemoattractant protein 1 (MCP-1) [149,150]. Notably, NF-κB can also indirectly inhibit SIRT1 activity through the regulation of multiple intermediating factors, forming a negative feedback loop [142]. In addition, SIRT1 provides protection against inflammatory injury partly via inhibition of the Nod-like receptor pyrin domain-containing protein 3 (NLRP3) inflammasome pathway [151].

SIRT1 also plays a crucial role in the regulation of cell survival. Through activation of the phosphoinositide 3-kinase/protein kinase B (PI3K/Akt) signaling pathway, SIRT1 inhibits apoptosis and enhances cell viability [152,153,154]. Its anti-apoptotic effects are also mediated by suppression of p53, a key regulator of cell cycle arrest and apoptosis [155]. Conversely, in hyperproliferative cells, SIRT1 can limit excessive proliferation and promote apoptosis [23,34,156], indicating that its effects on cell death are highly context- and cell type-dependent.

Beyond cell survival, SIRT1 influences pathological tissue remodeling. Overexpression or activation of SIRT1 suppresses hypoxia-inducible factor-1 (HIF-1) signaling, thereby attenuating cardiac hypertrophy [157]. SIRT1 also interacts with PPARα, promoting fatty acid oxidation and metabolic homeostasis. The SIRT1–PPARα complex can bind to NF-κB, inhibiting its signaling and preventing hypertrophic responses [158]. Importantly, SIRT1 exerts potent anti-fibrotic effects. Activation of SIRT1 inhibits the tissue growth factor β1/Small Mothers Against Decapentaplegic 3 (TGF-β1/Smad3) signaling pathway and attenuates fibrotic remodeling [159]. Mechanistic studies indicate that SIRT1 interacts with Smad2 independently of TGF-β stimulation, an effect that is enhanced in the presence of TGF-β [160], and deacetylates Smad4, further suppressing TGF-β/Smad signaling [144]. Additionally, SIRT1 activation reduces connective tissue growth factor (CTGF) expression, a downstream mediator of TGF-β signaling [63]. SIRT1 also counteracts fibrosis through inhibition of HIF-1–dependent TGF-β activation and via deacetylation of HIF-2α [159,161]. SIRT1 activity further modulates epithelial–mesenchymal transition (EMT), a process driven by TGF-β and characterized by increased fibronectin and α-smooth muscle actin (α-SMA) expression [144]. Activation of SIRT1 attenuates EMT, as evidenced by reduced levels of TGF-β, fibronectin, and α-SMA [162]. Moreover, a substantial proportion of fibroblasts in fibrotic tissue originate from endothelial-to-mesenchymal transition (EndMT), another TGF-β–dependent process. SIRT1 activation suppresses TGF-β/Smad2/3 signaling, thereby limiting EndMT and fibrotic progression [163,164,165]. Collectively, the findings demonstrate that SIRT1 activation prevents collagen deposition, whereas SIRT1 deficiency is associated with dysregulated extracellular matrix remodeling, including altered expression of collagen and MMPs [164,165,166].

SIRT1 plays a central role in regulating mitochondrial biogenesis and morphology. One of its main substrates, PGC-1α, is a key transcriptional coactivator that promotes mitochondrial biogenesis and maintains mitochondrial function [131,167]. SIRT1 also interacts with AMPK to modulate mitochondrial activity [167,168]. Interestingly, some studies suggest that mitochondrial biogenesis depends more on SIRT1 activity than on its protein abundance [167]. An imbalance between mitochondrial fusion and fission negatively affects mitochondrial function. Fusion is primarily regulated by outer mitochondrial membrane proteins, mitofusin 1 and 2 (Mfn1/2), whereas fission is controlled by dynamin-related protein 1 (Drp1). Activation of SIRT1 has been shown to increase Mfn2 expression and decrease Drp1 levels, thereby restoring mitochondrial dynamics and function [17,53].

## 4. Sirtuin 1 in Hypertension

### 4.1. Expression in Rodents

Numerous studies have reported downregulation of SIRT1 levels in the heart [83,169,170], aorta [29,136], mesenteric arteries (MAs) [156,171], kidneys [172,173], brain, including paraventricular nucleus (PVN) [30,174,175], cerebral cortex [149] and rostral ventrolateral medulla (RVLM) [150], and placenta [176,177] of hypertensive models. However, several studies reported increased SIRT1 expression in the heart [178,179], aorta [140,179,180,181], MAs [181] and kidneys [182]. A similar discrepancy was observed in the in vitro models: while most of the studies showed reduced SIRT1 levels in HUVECs [137,141,183], Hk2 [184], H9c2 [170,185], HL-1 [186], PC12 [150], NRK-52E [172], and HTR-8/SVneo [187] cells, one study reported increased SIRT1 expression [182]. The reasons for these conflicting results remain unclear and are unlikely to be explained by differences in rodent age, experimental models or Ang II concentration. Therefore, rather than being consistently downregulated, SIRT1 expression appears to be highly context-dependent, varying according to the pathological stimulus, experimental model, and type of intervention. This issue needs further investigation. Moreover, Luo et al. [171] demonstrated that exercise modulates not only SIRT1 level but also SIRT3, highlighting the broader importance of sirtuins in the pathophysiology of hypertension.

### 4.2. Effects in Rodents

The literature is clearly dominated by the spontaneously hypertensive rats (SHR) genetic model, whereas other models are represented by only a few individual studies. This limits the ability to generalize the effects of SIRT1 to all mechanisms underlying hypertension.

In SHR, SIRT1 activation or interventions that boost SIRT1 levels are generally associated with a reduction in BP and improvements regarding oxidative stress, inflammation, apoptosis, and cardiac or vascular remodeling. This pattern is observed across central, vascular and cardiac interventions [83,84,149,150,169,174,178]. A similar trend is seen in models of secondary hypertension, including Ang II-induced, 2K1C, arsenic-induced, CsA-induced, and salt-sensitive hypertension, as well as pre-eclampsia models [29,30,156,170,173,175,176,177,180,182]. However, the underlying mechanisms and predominant effects are model-specific, for instance, in SHR, a vast amount of data focuses on the neurohumoral/CNS axis, the heart and the vasculature, whereas in pre-eclampsia and pregnancy, the kidney, placenta, oxidative stress and angiogenesis play the dominant roles [173,176,177]. Antihypertensive effects of SIRT1 activation appear broadly conserved across experimental models, whereas the downstream mechanisms and target organs are strongly model dependent.

**Figure 3:**
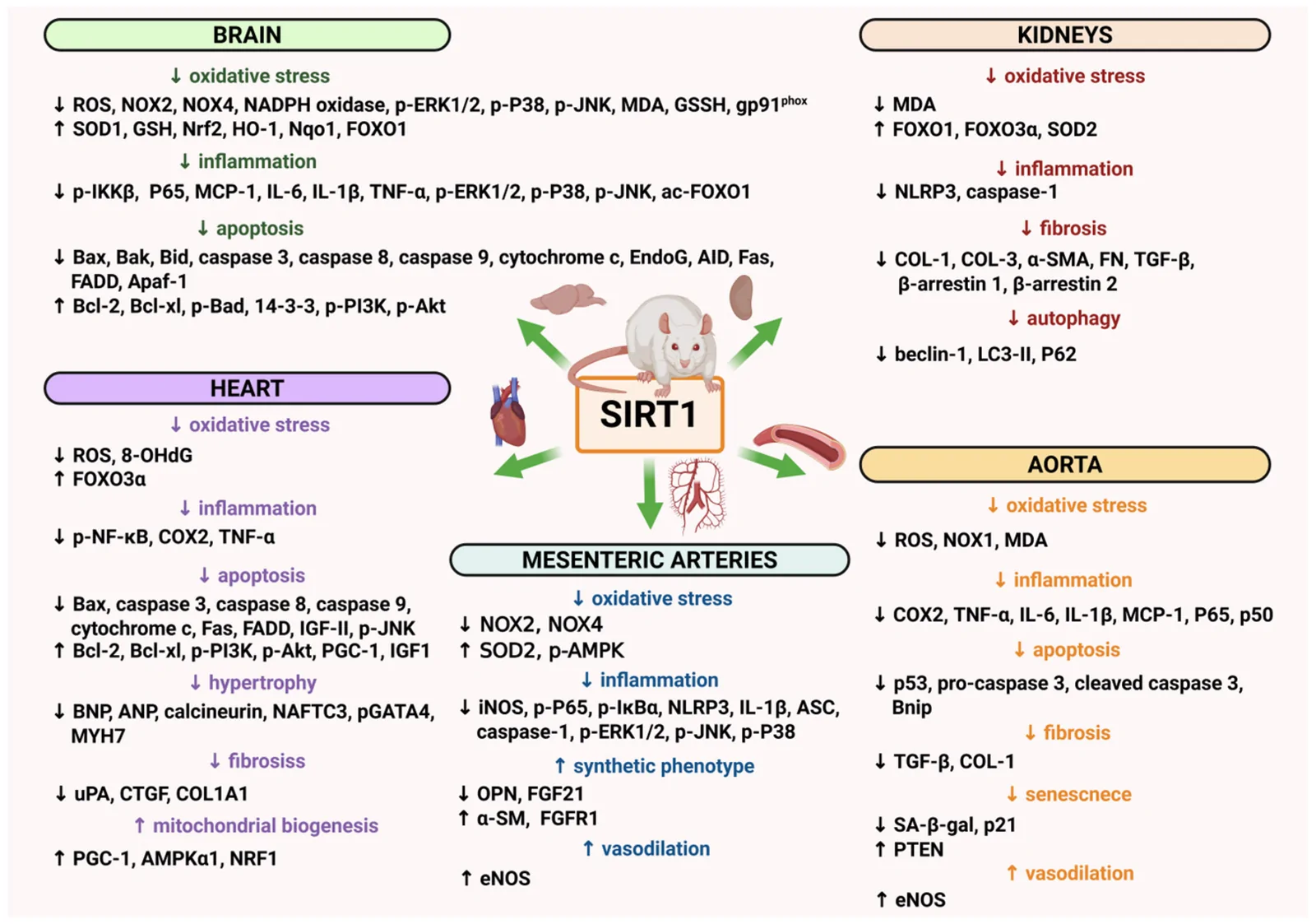
Molecular evidence of the biochemical changes in various organs after SIRT1 activation in hypertension in vivo. Created in BioRender.com.

In studies investigating the role of SIRT1 in hypertension, researchers predominantly administered natural modulators of this deacetylase, demonstrating multiple beneficial therapeutic effects (Table 2 and Table 3, Figure 3). Direct approaches to increase SIRT1 include lenti-SIRT1 [150] and recombinant SIRT1 [177]. Approaches that inhibit SIRT1 are much less common and primarily include EX527 [136,179] and NAM [175]. However, the paper of Cui et al. [136] is particularly valuable, because the effects of Pinggan-Qianyang decoction (PGQYD) on BP, oxidative stress, senescence, etc., is reversed by EX527, providing a stronger argument for the involvement of SIRT1 than the mere increase in its expression. An even stronger argument is provided by Niu et al. [175]: genistein and NAM combination leads to changes in oxidative stress and inflammation in the PVN that are opposite in direction to those observed with geniposide alone. It is important that natural compounds are not equivalent to the specific activation of SIRT1. Therefore, while the positive effects of substances such as REV or epigallocatechin-3-gallate (EGCG) are consistent with the involvement of SIRT1, they do not in themselves prove that SIRT1 is essential.

SIRT1 activators presented ability to decrease mean arterial pressure (MAP), SBP and DBP [29,30,83,84,136,149,150,156,169,170,171,174,175,176,177,178,180,182,188]. In one study, however, VH4 (Valine–Histidine–Valine–Valine) peptide treatment reduced DBP without affecting SBP [83]. Notably, two studies reported that higher doses of the administered compound significantly improved BP parameters, whereas lower doses were ineffective [156,176], indicating a dose-dependent effect that should be considered when evaluating therapeutic efficacy. In several studies, SIRT1 activation additionally reduced heart rate (HR) [84,149,169,171,174], while others reported no changes in HR [29,150,182]. Thus, the influence of SIRT1 on heart rate remains inconclusive.

In the model of primary hypertension—SHR—administration of irbesartan prior to the full consolidation of hypertension attenuated the progressive rise in BP, resulting in significantly lower final values compared to untreated age-matched controls [178]. The MAP, SBP and DBP of hypertensive rats decreased by approximately 60, 65 and 58 mmHg, respectively, following treatment. Significant BP reductions were also reported in SHR with severe hypertension treated with peptides IF and DF, with even greater effects observed when DF treatment was combined with exercise; however, the authors did not provide precise data [84,169]. Moderate alteration in BP was seen in rodents administered the peptide VH4, where MAP and DBP decreased by approximately 31 and 33 mmHg, respectively [83]. Similarly, treatment with flavonoid EGCG resulted in a decrease in MAP by 19 mmHg, SBP by 20 mmHg, and DBP by 18 mmHg [149]. Moderate effects were also noted following treatment with the Chinese medicine, PGQYD; however, no precise data were provided [136]. Smaller BP reductions, comparable to those reported by Hsieh et al. [149], were observed in SHR with less severe hypertension. Intracerebroventricular infusion of REV reduced MAP by 17 mmHg, similar to the effects observed after injection of SIRT1-overexpressing vectors into the RVLM of SHR [150]. Lower alteration in MAP was also noted after PVN infusion of capsaicin, although detailed data were not reported [174]. Physical activity also contributed to BP modulation. Low-intensity exercise (14 m/min) reduced MAP, SBP and DBP approximately by 18, 12 and 17 mmHg, respectively. Moderate-intensity exercise (20 m/min) resulted in greater reductions in MAP and SBP (by 30 and 24 mmHg, respectively), with a similar value of DBP. In contrast, high-intensity (26 m/min) training increased MAP by 8 mmHg and DBP by 6 mmHg, without affecting SBP [171]. Notably, the most pronounced BP reductions were observed in studies with treatment durations of 8 weeks [83,84,169,178]. These findings suggest that SIRT1-mediated hypotensive effects may depend on both the severity of hypertension and the duration of treatment. However, further studies including groups with varying disease severity and treatment duration are needed to confirm these observations.

Studies investigating the role of SIRT1 in secondary hypertension generally do not provide precise BP data, with the exception of two reports ([177,182]), and the outcomes vary across different experimental models. Based on approximate data, more severe hypertension appears to be associated with greater reductions in SBP and DBP in mice with Ang II-induced hypertension treated with SIRT1 activators such as a Chinese herbal medicine, Qianyang Yuyin granule (QYYYG) or a natural xanthone, α-mangostin (α-MG) [29,170]. Moreover, a higher dose of QYYYG produced a greater reduction in DBP compared to a lower dose (*p* < 0.001 vs. *p* < 0.01) [170]. Overall, further studies providing detailed data are required to better define the impact of SIRT1 activation on BP in models of secondary hypertension.

Importantly, SIRT1 contributed to increased vasodilatory responses to acetylcholine in aortas of SHR after running exercise [171], Wistar rats with arsenic-induced hypertension following melatonin treatment [180] and mice with Klebsiella pneumoniae-induced hypertension after incubation with REV [137]. However, melatonin did not change the vasodilatory response to sodium nitroprusside (SNP) [180]. SIRT1 activation also improved the vasodilatory response to PD166866 in MAs of SHR after swimming exercise and decreased the contractile response to NE [188]. Moreover, REV treatment resulted in reduced U46619-mediated vasoconstriction in MAs of rats with cyclosporine A-induced hypertension [156]. Melatonin administration decreased the contractile response to phenylephrine (Phe), but not to KCl in the aorta of Wistar rats with arsenic-induced hypertension [180]. Furthermore, co-incubation of the SHR aorta with the SIRT1 inhibitor EX527 and empagliflozin weakened the potency of acetylcholine-mediated vasorelaxation but did not affect the maximum response, whereas in MAs, SIRT1 inhibition significantly reduced maximum relaxation response to acetylcholine without influencing potency. In MAs of SHR, it also increased U46619-mediated vasoconstriction [181]. These findings suggest that SIRT1 is an important regulator of endothelial function, with its effects involving predominantly the NO/eNOS pathway. However, the available evidence also indicates that the contribution of SIRT1 may depend on the experimental model and the specific component of vascular function being assessed.

SIRT1 activation alleviates structural alterations in tissues of hypertensive rodents. It reduces or prevents cardiac hypertrophy [84,169,170,174], as evidenced by decreased levels of hypertrophic markers such as brain natriuretic peptide (BNP) and atrial natriuretic peptide (ANP). Additionally, SIRT1 suppresses calcineurin/nuclear factor of activated T cells 3 (NFATC3) signaling [84,169], a pathway implicated in CVD development [189]. Reduced aortic wall thickness following SIRT1 stimulation has also been reported [136,171]. Furthermore, SIRT1 exerts protective effects against fibrotic remodeling in the heart [84] and kidneys [172,182], primarily through the reduction in collagen deposition and inhibition of the pro-fibrotic TGF-β1 signaling pathway. A common mechanistic axis links vascular, cardiac, and renal pathologies in hypertension: SIRT1 acts as a central hub regulating ROS production, NF-κB-mediated low-grade inflammation, and fibrotic extracellular matrix (ECM) deposition across these tissues. Specifically, SIRT1 upregulation suppresses key ECM and pro-fibrotic proteins, notably collagen type I (COL1A1), collagen type III (COL3A1), CTGF, and TGF-β1, thereby reducing vascular stiffness, renal fibrosis, and cardiac hypertrophic remodeling [84,169,172,182].

In studies evaluating cardiac remodelling, SIRT1 activation—promoted by pharmacological agents (VH4, irbesartan, QYYYG, capsaicin, IF, DF)—consistently attenuates cardiac hypertrophy and fibrosis [83,84,169,170,174,178]. These interventions reduce heart weight, left ventricular mass index, cardiomyocyte diameter, and hypertrophic markers (BNP, ANP), while suppressing pro-fibrotic collagen deposition and modulating calcineurin/NFATC3/pGATA4 signalling [83,84,169,170,174,178]. Consequently, SIRT1 engagement enhances structural integrity, preserves mitochondrial biogenesis, and improves functional parameters like fractional shortening, establishing the attenuation of cardiac remodelling as a major tissue-specific effect of SIRT1 activation. In some in vitro models, increased SIRT1 activity or expression promoted proliferation and migration [51,185], whereas SIRT1 inhibition suppressed these processes [51,140]. However, this relationship was not uniform across all cell types and experimental conditions.

SIRT1 also contributes to the maintenance of tissue integrity by suppressing apoptosis in hypertension. Anti-apoptotic effects have been observed in the heart [83,84,169,178], aorta [136,180], and cerebral cortex [149]. These effects were associated with increased expression of anti-apoptotic proteins (B-cell lymphoma 2 (Bcl-2), B-cell lymphoma-extra large (Bcl-xl)) in the heart and brain and reduced levels of pro-apoptotic markers, including Bcl-2-associated X protein (Bax), cytochrome c (cyto c), caspase 3, caspase 8, and caspase 9. Mechanistic studies highlighted the involvement of the PI3K/Akt pathway, particularly phosphorylated forms, in the regulation of mitochondrial-dependent apoptosis in the heart and brain of SHR [83,84,149,169]. The mitochondrial apoptotic pathway is one of the key pathways that regulates cell death and it is controlled by Bcl-2 family proteins. The signal transduction through the mitochondrial membrane ultimately leads to caspase 3 activation and induction of apoptosis [190]. Activation of SIRT1 cooperates with AMPK to inhibit hyperactive mTOR and simultaneously activate the PI3K/Akt axis. Phosphorylation of PI3K/Akt prevents apoptosis by phosphorylating Bcl-2-associated death promoter (Bad), thereby inhibiting its interaction with Bcl-2 [191] and promoting its binding to the 14-3-3 protein [192]. It results in the upregulation of anti-apoptotic Bcl-2 and Bcl-xl, while downregulating pro-apoptotic Bax, cyto c release, and caspase activation. Through this crosstalk between mTOR suppression and Akt/Bcl-2 activation, SIRT1 preserves mitochondrial survival and prevents apoptotic cell death in cardiomyocytes, vascular cells, and neural tissue under hypertensive conditions.

Furthermore, in an in vitro study using a cellular model of Ang II–induced hypertension, treatment with bazedoxifene resulted in an increase in the anti-apoptotic protein Bcl-2 and a decrease in the levels of pro-apoptotic Bax and cleaved caspase-3 [141]. Similarly, geniposide treatment in a high-salt model led to elevated levels of Bcl-2 and the phosphorylated form of AMPK, along with reduced levels of Bax and phosphorylated p38 kinase, indicating anti-apoptotic properties on the cardiac cells [185].

Although not evaluated across all tissue models, anti-apoptotic activity represents a highly recurrent downstream protective effect of SIRT1 activation. In cardiac models, interventions such as exercise, DF, IF and irbesartan activate the IGF1/PI3K/Akt survival axis and upregulate anti-apoptotic proteins (Bcl-2, Bcl-xL) [83,84,169,178]. Concurrently, SIRT1 engagement suppresses pro-apoptotic cascades, reducing Bax, cyto c release, caspases-3/8/9, and the type I integral membrane protein/Fas ligand/Fas-associated protein with death domain (Fas/FasL/FADD) pathway to mitigate cell death and structural remodelling [178]. Mechanistically, these anti-apoptotic outcomes align with SIRT1’s established regulation of p53 and FOXO-mediated cell survival during oxidative stress.

SIRT1 modulation was generally associated with reduced apoptotic signaling in vitro, as reflected by decreased expression of pro-apoptotic markers [141,183,185] and increased expression of anti-apoptotic Bcl-2 [141,185]. However, the evidence was limited to selected experimental models, and the strength of the association varied depending on the model and the experimental approach.

SIRT1 stands out for its ability to attenuate oxidative stress and inflammation in hypertension throughout the body. In several experimental models, reduced SIRT1 expression was accompanied by increased oxidative stress, reflected by higher ROS, superoxide, malondialdehyde (MDA), or NOX activity. This was observed, for example, in Ang II-induced endothelial dysfunction [141,183]. Excessive production of ROS, a hallmark of oxidative stress, was reduced following treatment with SIRT1 activators in the brain [30,150,174,175] and heart [170], thereby limiting further oxidative tissue damage. ROS were also reduced in the cell culture models after treatment with trans-resveratrol [193], bazedoxifene [141] and when overexpression of SIRT1 was induced [186,187]. NOX are among the principal sources of ROS overproduction and contribute to vascular dysfunction in hypertension [194]. Importantly, SIRT1 activation and overexpression significantly reduced the expression of NOX2 [137,140,171,174], and NOX4 [30,171] in both in vivo and in vitro studies. Unexpectedly, one study reported that the SIRT1 inhibitor EX527 exerted antioxidative effects by reducing NOX2 and NOX4 expression in VSMCs of SHR [179]. This paradoxical finding warrants further investigation to clarify the role of SIRT1 in oxidative stress regulation in rodent models of primary hypertension. Another key pathway mediating the antioxidative effects of SIRT1 in hypertension is the Nrf2/HO-1 axis [195,196]. HO-1 has been shown to protect against Ang II–induced oxidative stress [197] and to exert hypotensive and vasodilatory effects in MAs of SHR [198], underscoring its pivotal role in hypertension. SIRT1 activators increased Nrf2 and HO-1 expression in the PVN [175] and aorta [180] of hypertensive rodents, but also in the in vitro studies on cardiac cells [186] and HUVECs [183]. Moreover, Nrf2 activation enhances the transcription of additional antioxidant factors, including SOD and GSH, via binding to antioxidant response elements (ARE) [195]. Consistently, SIRT1 modulation increased SOD levels in the aorta [136], brain [30,150,175], serum [171], placenta in gestational hypertension (GH) models [176], and protected kidneys against hypertensive injury [172,173]. SOD also increased in HL-1 cell culture (cardiac muscle cell line) overexpressing SIRT1 [186], as well as in the HTR-8/SVneo cells (a trophoblast cell line) after treatment with alpinumisoflavone (AIF) [51], in the rat kidney epithelial cell line (NRK-52E cells) treated with REV or *C. cicadae* [172] and PC12 cell culture (derived from rat pheochromocytoma) incubated with REV [150], [Table 4 and Table 5]. Similarly, GSH levels were significantly elevated in the aorta [180], PVN [175], serum [171], and placenta [176] following treatment with natural SIRT1 activators or exercise interventions. SIRT1 activation effectively increased GSH also in HL-1 cells stimulated with high salt concentration [186]. Overall, these findings suggest that SIRT1 is consistently linked to the regulation of oxidative stress, while changes in individual antioxidant markers appear to be more context-dependent.

The SIRT1-mediated upregulation of antioxidant enzymes, including SOD and GSH, serves as a fundamental protective mechanism against hypertensive vascular dysfunction [199]. While these antioxidant responses have been observed across various cell types and tissue beds—including the aorta [136], cerebral tissue [30,150,175], placenta [176], and renal epithelium [172,173]—their mechanistic relevance is fundamentally rooted in vascular ECs and VSMCs. In the vascular wall, increased SOD activity directly scavenges superoxide anions, thereby preventing the rapid quenching of NO and the formation of cytotoxic peroxynitrite [200]. Concurrently, elevated GSH levels preserve intracellular redox balance, protecting endothelial eNOS from uncoupling and suppressing ROS-induced VSMC proliferation [201]. Thus, the modulation of SOD and GSH in non-vascular models mirrors a conserved SIRT1-driven antioxidant network that is directly essential for preserving vascular tone and preventing microvascular damage in hypertension.

Activation of SIRT1 consistently attenuates oxidative stress across diverse tissues by suppressing ROS production and enhancing antioxidant defenses [30,170,174,175]. In the central nervous, CV, renal, placental, and myocardial systems, SIRT1 activation—driven by compounds such as REV, genistein, or melatonin, as well as exercise—downregulates NOX activity and reduces markers of oxidative damage, including MDA, 3-nitrotyrosine (3-NT), and 8-hydroxy-2′-deoxyguanosine (8-OHdG) [30,136,170,171,172,176,180]. Concurrently, SIRT1 activation upregulates SOD, GSH, and key cytoprotective pathways (Nrf2/HO-1, FOXO3a), reinforcing SIRT1’s role as a master regulator of redox homeostasis [30,136,171,172,176,180]. In the in vitro studies, SIRT1 modulation was linked to mitochondrial homeostasis, with reported effects on mitochondrial integrity, energy metabolism, and cellular responses to oxidative and metabolic stress [51,183,186].

If we observe an increase in SIRT1 and oxidative stress in the context of hypertension, this could theoretically be interpreted as an attempt to counteract oxidative stress. However, to classify this as a compensatory response, we would need, for example, a longitudinal study showing that SIRT1 levels rise after the onset of hypertension, and that this increase subsequently limits the further progression of the disease; or a scenario where blocking this increase worsens the phenotype.

MAPK proteins constitute a crucial signaling pathway involved in the pathophysiology of hypertension [202] and play a well-established role in the regulation of oxidative stress and inflammatory responses. SIRT1 has been shown to reduce the phosphorylation of MAPK kinases, including ERK1/2, JNK, and p38, thereby attenuating oxidative stress and inflammation in the PVN of SHR [174]. Similarly, REV-mediated activation of SIRT1 reduced MAPK-dependent inflammatory responses in MAs of hypertensive rats [156]. Hypertension is increasingly recognized as an immune-mediated disease. Monocytes from hypertensive patients secrete elevated levels of pro-inflammatory cytokines, including TNF-α, IL-1β, and IL-6 [203]. Consistently, cytokine levels were higher in hypertensive animal models than in rodents treated with SIRT1 activators or SIRT1 overexpression vectors [174,175,177]. Activation of SIRT1 by capsaicin or geniposide effectively reduced interleukin and TNF-α levels in the PVN [174,175], while the anti-inflammatory effects of α-mangostin (α-MG) were confirmed in the aorta of mice with Ang II–induced hypertension [29]. Monocyte recruitment is driven by the chemokine MCP-1, whose expression is closely associated with ROS production. Following differentiation into macrophages, these cells release inflammatory cytokines and exacerbate endothelial dysfunction [204]. MCP-1 levels are elevated in hypertension and are considered a key contributor to disease pathophysiology. Notably, SIRT1 activation significantly reduced MCP-1 expression in the PVN [174], underscoring the role of SIRT1 in modulating vascular inflammation. Another central mediator of immune activation in hypertension is NF-κB. Excessive ROS production promotes NF-κB activation, leading to increased transcription of pro-inflammatory genes [205]. SIRT1 activators were shown to reduce NF-κB expression in the PVN [30,174] and aorta [29], thereby attenuating inflammation in hypertension. Moreover, polysaccharides from Atractylodes macrocephala Koidz (PAMK) effectively reduced inflammation in an Ang II–stimulated kidney cell line by lowering key pro-inflammatory cytokines, such as TNF-α and IL-1β, through SIRT1 signaling [182].

SIRT1 activation consistently suppresses inflammation across central, cardiac, and vascular tissues by inhibiting the NF-κB signaling pathway [30,84,174]. Centrally in the PVN, as well as peripherally in the heart and vasculature, SIRT1 activation—triggered by agents like capsaicin, genistein, REV, melatonin, or exercise—downregulates pro-inflammatory cytokines (IL-1β, IL-6, TNF-α), COX-2, and NLRP3 inflammasome components [30,84,171,174,175,180]. Mechanistically, this anti-inflammatory effect is driven primarily by SIRT1-mediated deacetylation of the NF-κB p65 subunit, establishing inflammatory suppression as a core outcome of SIRT1 activation. SIRT1 activation or overexpression in the in vitro studies was also frequently associated with suppression of inflammatory signaling, including NF-κB/NLRP3-related pathways [137,182,187].

Chronic hypertension disrupts mitochondrial function and impairs mitochondrial biogenesis. In addition, cellular senescence promotes mitochondrial damage and facilitates hypertensive injury in the heart [206]. Several studies have shown that SIRT1-activating peptides, including VH4, IF, and DF, enhance mitochondrial biogenesis primarily through upregulation of PGC-1α [83,84,169]. Similarly, Chinese herbal medicine increased PGC-1α expression and improved mitochondrial integrity in the heart of mice with Ang II-induced hypertension, as well as in the cardiac cell line stimulated with Ang II [170]. In HTR-8/SVneo cells treated with AIF, mitochondrial function was improved through upregulation of genes encoding components of the mitochondrial electron transport chain (ETC), including mitochondrial complex I subunit-encoding genes (NDUFA2, and NDUFS7), complex III subunit-encoding genes (UQCRB, UQCRC1, and UQCRFS1), and complex V subunit-encoding genes (ATP5MC1, ATP5ME, and ATP5PD) [51]. The role of SIRT1 in aging attenuation is well established. In hypertensive models, SIRT1 activation was associated with reduced levels of the senescence marker, p21, a protein that initializes cell cycle arrest, as well as decreased senescence-associated β-galactosidase (SAβG) activity, which reflects age-related structural cellular changes [136,207]. Moreover, SIRT1 activation upregulated phosphatase and tensin homolog (PTEN) expression in the aorta, the loss of which is characteristic of cellular senescence [136].

There is no comparison of acute versus chronic SIRT1 activation within the same model that would allow one to state that the short-term effect differs qualitatively from the chronic one. The available evidence predominantly reflects chronic SIRT1 modulation [30,83,84,136,149,169,170,171,172,174,175,178,180,182], whereas acute effects and temporal dynamics of SIRT1 activation remain poorly characterized [29,150,156,179].

Although autophagy is essential for maintaining cellular homeostasis and delaying aging and is generally promoted by SIRT1 [131], studies included in this review reported reduced Beclin-1 levels following treatment with SIRT1 activators [172,180], a key initiator of autophagy [208]. Autophagy is commonly induced under stress conditions to supply energy substrates and support cell survival; however, excessive autophagy may result in the depletion of essential cellular components and ultimately trigger cell death [209]. These findings suggest that SIRT1 may act as either a positive or negative regulator of autophagy, depending on disease and the intensity of autophagy-related cell stress, although further investigation is required.

In addition to pharmacological SIRT1 activators, several studies evaluated the effects of exercise on SIRT1 expression and function. In one study, combined VH4 peptide treatment and swimming exercise improved cellular energy balance, as evidenced by increased AMPK levels [83]. In another study, swimming combined with IF administration resulted in greater improvement in ejection fraction compared with peptide treatment alone [84]. Interestingly, exercise intensity was shown to affect hypertensive rodents differently than previously assumed based on human studies. After 14 weeks of treadmill running, SHR in the low- and moderate-intensity groups (14 and 20 m/min) exhibited reduced BP and HR, whereas high-intensity exercise (26 m/min) increased MAP and DBP, with no changes in SBP or HR. The authors emphasized that the available data were insufficient to explain why high-intensity interval training in humans and high-intensity continuous training in this rat model produced opposite effects on BP [171]. It should be noted that in clinical settings, physical exercise acts as part of a comprehensive lifestyle modification alongside weight reduction, dietary adjustments, and improved sleep hygiene. In animal models, the impact of exercise on BP is highly protocol-dependent and varies depending on the intensity of the training.

SIRT1 is being studied in a wide variety of localizations, but it is not simply a single, universal SIRT1 mechanism. In the PVN/RVLM, effects are associated with ROS/NOX, FOXO1, NF-κB, neurotransmission and sympathetic activity [30,150,174,175]. In the heart, PI3K/Akt, FOXO3α, AMPK/PGC-1α, mitochondria, apoptosis, fibrosis and hypertrophy predominate [83,84,169,178]. In blood vessels/VSMCs, NOX2/NOX4, SOD, eNOS, iNOS, NF-κB, nitrosative stress, and endothelial function are involved [149,179], while a study of Balarastaghi et al. [180] focused on oxidative stress, the Nrf2/HO-1 response, and aortic damage. In the kidneys, SIRT1 is linked to protection against renal injury, with additional effects involving oxidative stress [172,173], fibrosis [172,182] and autophagy [172]. This provides strong grounds for arguing that SIRT1 acts in a tissue-specific manner rather than through a single common downstream pathway.

SIRT1 was modulated predominantly through systemic administration, most commonly by the oral/i.g. route [29,84,136,149,156,169,170,173,176,182], while a smaller but mechanistically distinct group of studies used direct central administration into the PVN [30,174,175] or RVLM [150,176] or intracerebroventricular (ICV) infusion [150]. Central approaches were specifically used to investigate SIRT1-dependent regulation of autonomic, oxidative and inflammatory mechanisms within the brain [30,150,174,175,176], whereas systemic administration was associated with effects observed in peripheral organs including the heart, aorta, kidneys and placenta [29,83,84,136,149,156,169,170,172,173,176,178,179,180,182].

The available evidence is strongly male-biased, while female animals have been studied predominantly in pregnancy-related hypertension models [173,176,177]. Importantly, none of the studies directly investigated sex-dependent differences in SIRT1-mediated regulation of BP.

The studies encompass a relatively wide age range, extending from young animals, e.g., 4 weeks of age [175], through 8–12 weeks [29,83,84,169,170,171,172,173,174,176,178,179,182] and 16–18 weeks [150,177], up to 36 weeks [149] and 64 weeks [136]. However, none of these studies systematically investigated the age dependence of SIRT1-mediated effects. Although a study on 64-week-old SHRs [171] linked SIRT1 to vascular aging processes, it did not directly evaluate an age-dependent response.

**Table 2:** Effects of SIRT1 activation on hypertension: evidence from in vivo studies.

Species/Sex/ Age/Weight	Model	Modulator	Effective Dose/Concentration, Duration/Time and Route of Administration		SIRT1 *	Effects						Ref.
						SIRT1	BP, HR	Anti- Oxidative	Anti- Inflammatory	Anti- Apoptotic	Others	
Primary Hypertension												
Preventive												
SHR offspring male 21 days of embryonic period n/d	SHR	swimming exercises	20 min/day, increased by 10 min/day until reached 60 min/day 6 days/week during pregnancy		n/d	↑ in MAs	n/d	n/d	n/d	n/d	in MAs: switch to synthetic phenotype (↓ OPN, FGF21, ↑ α-SM, FGFR1) ↓ H3K9ac	[188]
SHR offspring male 3 months n/d						n/d	↓ SBP, DBP, MAP	n/d	n/d	n/d	in MAs: switch to synthetic phenotype (↓ OPN, FGF21, ↑ α-SM, FGFR1) ↑ lumen diameter, vasodilatory response to PD166866 ↓ WT, wall/lumen ratio, contractile response to NE	
SHR male 8 weeks n/d	SHR	irbesartan	50 mg/kg 8 weeks s.c.		↑	↑ in LV	↓ SBP, DBP, MAP			in LV: ↑ Bcl2, Bcl-XL, PGC-1 ↓ Bax, cyto c, activated caspase 3, activated caspase 8, activated caspase 9, Fas L, Fas, FADD, IGF-II, p-JNK ↓ apoptosis in assay	↓ HW, IVSd ↔ BW in LV: ↑ FS ↓ LVW, LVIDs ↔ LVPWd, LVIDd	[178]
therapeutic												
SHR male 12 weeks n/d	SHR	capsaicin	2 μg/h 28 days PVN infusion		↓	↑ in PVN	↓ MAP, HR	in PVN: ↓ ROS, NOX2, NADPH oxidase, p-ERK1/2, p-P38, p-JNK	in plasma: ↑ IL-10 ↓ IL-1β in PVN: ↓ p-IKKβ, NF-κB P65, MCP-1, IL-6, IL-1β, TNFα, p-ERK1/2, p-P38, p-JNK	n/d	↓ ACE, AT1R, TH in PVN: ↑ inhibitory neurotransmitters production (↑ GAD67) in plasma: ↓ NE, EPI in heart: ↓ Hypertrophy (↓ ANP, BNP), LV cardiomyocytes diameter	[174]
SHR male 36 weeks n/d	SHR	TEAVIGO (EGCG 94%)	200 mg/kg 12 weeks orally		↓	↑ in cerebral cortex	↓ HR, SBP, DBP, MAP, PP	n/d	n/d	in cerebral cortex: ↑ Bcl-2, Bcl-xl, p-Bad, 14-3-3, p-PI3K, p-Akt ↓ Bax, Bak, caspase-3, caspase-8, caspase-9, cyto c, EndoG, AIF, FasL, Fas, FADD, t-Bid, Apaf-1 ↓ in assay ↔ Bad	↔ BW, brain weight	[149]
SHR male 16–18 weeks 280–350 g	SHR	REV	250 μg 7 days ICV infusion		↓	n/d	↓ MAP ↔ HR	n/d	n/d	n/d	↓ RSNA, NE in 24h-urine	[150]
		lenti-SIRT1	0.2 μL RVLM injection			↑	↓ MAP ↔ HR	in RVLM: ↑ SOD1, FOXO1 ↓ ROS	n/d	n/d	↓ RSNA	
SHR n/d 12 weeks n/d	SHR	VH4	25 mg/kg 8 weeks i.p. swimming for 20 min in 1st week, 40 min in 2nd week, 60 min in 3–8th weeks		↓	↑ in heart	↓ HR, MAP, DBP ↔ SBP	n/d	n/d	in heart: ↑ Bcl2, Bcl-XL, p-Akt, p-PI3K	in heart: ↑ FS, p-FOXO3α, mitochondrial biogenesis (↑ PGC1α) ↓ HW ↔ EF, cellular energy balance (↔ AMPK)	[83]
		exercises				↑ in heart	↓ HR, MAP, DBP ↔ SBP	n/d	n/d	in heart: ↑ Bcl-XL, p-PI3K, ↔ Bcl2, p-Akt	in heart: ↑ FS, EF, p-FOXO3α, mitochondrial biogenesis (↑ PGC1α) ↓ HW ↔ cellular energy balance (↔ AMPK)	
		VH4 + exercises				↑ in heart	↓ HR, MAP, DBP ↔ SBP	n/d	n/d	in heart: ↑ Bcl2, Bcl-XL, p-Akt, p-PI3K	in heart: ↑ FS, EF, p-FOXO3α, mitochondrial biogenesis (↑ PGC1α), cellular energy balance (↑ AMPK) ↓ HW	
SHR male 12 weeks n/d	SHR	IF	10 mg/kg 8 weeks i.g. swimming for 20 min in 1st week, 40 min in 2nd week, 60 min in 3–8th weeks		↔	↑ in heart	↓ HR, SBP, DBP, MAP	n/d	in heart: ↓ p-NF-κB, COX-2, IL-6, TNFα	in heart: ↑ IGF1, p-Akt, p-PI3K	in blood: ↓ CK, LDH, AST, creatinine ↔ T-CHO, TG, HDL, LDL-C, ALT, uric acid in heart: ↑ mitochondrial biogenesis (↑ AMPKα1, PGC1α) ↓ HW, fibrosis (↓ uPA, CTGF, COL1A1), hypertrophy (↓calcineurin, NAFTC3, pGATA4, BNP) ↔ FS, EF	[84]
		exercises				↑ in heart	↓ HR, SBP, DBP	n/d	in heart: ↓ p-NF-κB, IL-6 ↔ COX-2, TNFα	in heart: ↑ IGF1, p-Akt, p-PI3K	in blood: ↓ CK, LDH, AST, creatinine ↔ T-CHO, TG, HDL, LDL-C, ALT, uric acid in heart: ↑ FS, EF, mitochondrial biogenesis (↑ AMPKα1, PGC1α) ↓ HW, fibrosis (↓ uPA, CTGF, ↔ COL1A1), hypertrophy (↓ calcineurin, NAFTC3, pGATA4, BNP)	
		IF + exercises				↑ in heart	↓ HR, SBP, DBP	n/d	in heart: ↓ p-NF-κB, COX-2, IL-6, TNFα	in heart: ↑ IGF1, p-Akt, p-PI3K	in blood: ↓ CK, LDH, AST, creatinine ↔ T-CHO, TG, HDL, LDL-C, ALT, uric acid in heart: ↑ FS, EF, mitochondrial biogenesis (↑AMPKα1, PGC1α) ↓ HW, fibrosis (↓uPA, CTGF, COL1A1), hypertrophy (↓calcineurin, NAFTC3, pGATA4, BNP)	
SHR n/d 12 weeks n/d	SHR	DF	10 mg/kg 8 weeks i.g. swimming for 20 min in 1st week, 40 min in 2nd week, 60 min in 3–8th weeks		↓	↑ in heart	↓ HR, SBP, DBP, MAP	in heart: ↑ FOXO3α	n/d	in heart: ↑ Bcl-2, Bcl-xl, IGF1R, p-PI3K, p-Akt ↔ Bad, IGF1 ↓ apoptosis in assay	in serum: ↓ CK, LDH, AST, creatinine ↔ T-CHO, TG, HDL, LDL-C, ALT, uric acid in heart: ↑ EF, FS, mitochondrial biogenesis (↑AMPKα1, PGC1α) ↓ fibrosis (↓uPA, CTGF, COL1A1, COL3A1), hypertrophy (↓calcineurin, NAFTC3, BNP, ANP, ↔ pGATA4)	[169]
		exercises				↑ in heart	↓ HR, SBP, DBP, MAP	in heart: ↑ FOXO3α	n/d	in heart: ↑ Bcl-2, IGF1 ↔ Bcl-xl, Bad, IGF1R, p-PI3K, p-Akt ↓ apoptosis in assay	in serum: ↓ CK, LDH, AST, creatinine ↔ T-CHO, TG, HDL, LDL-C, ALT, uric acid in heart: ↑ EF, FS, mitochondrial biogenesis (↑AMPKα1, ↔ PGC1α) ↓ fibrosis (↓CTGF, COL1A1, ↔ uPA, COL3A1), hypertrophy (↓calcineurin, NAFTC3, BNP, ANP, ↔ pGATA4)	
		DF + exercises				↑ in heart	↓ HR, SBP, DBP, MAP	in heart: ↑ FOXO3α	n/d	in heart: ↑ Bcl-2, Bcl-xl, Bad, IGF1, IGF1R ↔ p-PI3K, p-Akt ↓ apoptosis in assay	in serum: ↓ CK, LDH, AST, creatinine ↔ T-CHO, TG, HDL, LDL-C, ALT, uric acid in heart: ↑ EF, FS, mitochondrial biogenesis (↑AMPKα1, PGC1α) ↓ fibrosis (↓ uPA, CTGF, COL1A1, COL3A1), hypertrophy (↓calcineurin, NAFTC3, BNP, ANP, pGATA4)	
SHR male 8 weeks n/d	SHR	EX527	5 mg/kg twice/week for 3 weeks i.p.		↑	↓ in heart, aorta and VSMCs	in VSMCs: ↓ O_2_^−^, NADPH oxidase, NOX2, NOX4, FOXO1	n/d	n/d		in heart and aorta: ↓ Giα-2 ↑ eNOS in VSMCs: ↓ Giα-2, p47^phox^, nitrosative stress (↓ONOO^−^, n-tyr, ↑NO)	[179]
SHR male 64 weeks 230–250 g	SHR	PGQYD	0.2 g/mL, 1 mL/100 g 49 days i.g.		↓	↑ in aorta	↓ SBP	in aorta: ↑ anti-superoxide anion, SOD ↓ NADPH oxidase	n/d	in aorta: ↓ p53	in aorta: ↓ MT, MT/LD, senescence (↓SA-β-gal, p21, ↑ PTEN)	[136]
			0.2 g/mL, 1 mL/100 g 49 days i.g. + EX527 10 mg/kg i.p.			↓ in aorta	↑ SBP	in aorta: ↓ anti-superoxide anion, SOD ↑ NADPH oxidase	n/d	in aorta: ↑ p53	in aorta: ↑ MT, MT/LD, senescence (↑SA-β-gal, p21, ↓PTEN)	
SHR male 8 weeks 190–200 g	SHR	running on a treadmill	14 weeks 5 times/week 60 min	speed: 14 m/min	↓	↑ in MAs	↓ HR, MAP, SBP, DBP	in serum: ↑ GSH-Px, SOD ↓ MDA in MAs: ↑ SOD2, p-AMPK ↓ NOX2, NOX4	in MAs: ↓ iNOS, p-P65, p-IκBα, NLRP3, IL-1β, TLR4, ASC, caspase-1	n/d	↓ BW in aorta: ↑ vasodilatory response to Ach ↓ intima-media thickness, intima-media cross-sectional area in MAs: ↑ SIRT3, endothelial function (↑p-eNOS, eNOS) ↓ 3-NT	[171]
				speed: 20 m/min		↑ in MAs	↓ HR, MAP, SBP, DBP	in serum: ↑ GSH-Px, SOD ↓ MDA in MAs: ↑ SOD2, p-AMPK ↓ NOX2, NOX4	in MAs: ↓ p-P65, p-IκBα, NLRP3, IL-1β, TLR4, ASC, caspase-1	n/d	↓ BW in aorta: ↑ vasodilatory response to Ach ↓ intima-media thickness, intima-media cross-sectional area in MAs: ↑ SIRT3, endothelial function (↑p-eNOS, eNOS) ↓ iNOS, 3-NT	
				speed: 26 m/min		↔ in MAs	↑ MAP, DBP ↔ SBP, HR	in serum: ↔ GSH-Px, MDA, SOD in MAs: ↔ p-AMPK, SOD2, NOX2, NOX4	in MAs: ↓ iNOS, p-P65, p-IκBα, NLRP3, IL-1β, TLR4, ASC, caspase-1	n/d	↓ BW in aorta: ↓ vasodilatory response to Ach ↔ intima-media thickness, intima-media cross-sectional area in MAs: ↑ endothelial function (↑eNOS, ↓p-eNOS) ↔ SIRT3, 3-NT	
SHR male 8 weeks 170–210 g	SHR	REV	40 mg/kg 28 weeks i.p.		↓	↓ in kidneys	n/d	in kidneys: ↓ MDA ↑ FOXO3a, SOD	n/d	n/d	in kidneys: ↓ fibrosis (↓COL-1, α-SMA, ↔ FN, TGFβ1), autophagy (↓beclin-1, LC3-II, P62)	[172]
		*C. cicadae*	4 g/kg 28 weeks i.p.			↑ in kidneys		in kidneys: ↓ MDA ↑ FOXO3a, SOD	n/d	n/d	in 24h-urine: ↓ kidney injury (↓albumin, β2-MG, KIM-1) in kidneys: ↓ fibrosis (↓COL-1, α-SMA, FN, TGFβ1), autophagy (↓ beclin-1, LC3-II, P62)	
secondary hypertension												
preventive												
SD rats male n/d 275–300 g	2K1C	REV	160 μmol/L, 0.4 μL 4 weeks PVN infusion		↓	↑ in PVN	↓ SBP	in PVN: ↑ SOD1 ↓ NADPH oxidase, ROS, NOX4	in PVN: ↓ NF-κB P65	n/d	in plasma: ↓ NE in PVN: ↑ inhibitory neurotransmitters production (↑GAD67) ↓ TH	[30]
C57BL/6J mice male 8 weeks n/d	Ang II-induced (1 μg/kg/min, 28 days)	QYYYG	7.8 g/kg 4 weeks 3 days after pump implantation i.g.		↓	↑	↓ SBP, DBP	in heart: ↓ ROS, 8-OHdG	n/d	n/d	in heart: ↓ LVMI, collagen, hypertrophy (↓BNP, MYH7) ↑ LVEF, MV E/A, mtDNA, mitochondrial function (↑TFAM, TFB2M), mitochondrial biogenesis (↑PGC1α, NRF1)	[170]
			3.9 g/kg 4 weeks 3 days after pump implantation i.g.			↑ expression ↔ mRNA	↓ SBP, DBP	in heart: ↓ ROS, 8-OHdG	n/d	n/d	in heart: ↓ collagen, hypertrophy (↓BNP, MYH7) ↔ LVMI, MV E/A ↑ LVEF, mtDNA, mitochondrial function (↑TFAM, TFB2M), mitochondrial biogenesis (↑PGC1α, ↔ NRF1)	
C57BL/6 mice male 8 weeks n/d	Ang II-induced (500 ng/kg/min, 2 weeks of infusion)	α-MG	4 mg/kg, 0.2 mL 14 days, twice a day by gavage		↓	↑ in aorta	↓ SBP, DBP ↔ HR	n/d	in aorta: ↓ TNF, p65, p50, COX-2	n/d	↔ BW in aorta: ↑/↔ α-MG gene targets (↑ HSP90AA1, PPARG, ↔ AKT1, SRC, CTNNB1, HSP90AB1, mTOR, MAPK1, MAPK3, CCND1, BCL2L1, PIK3CA, HDAC1, PRKCA, APP)	[29]
Wistar male n/d 230–250 g	As-induced (15 mg/kg, 28 days, gavage)	Mel	10 mg/kg 28 days i.p.		↑	n/d	↓ SBP	in aorta: ↓ MDA ↑ GSH	n/d	n/d	in aorta: ↔ histopathological alterations	[180]
			20 mg/kg 28 days i.p.			↓ in aorta	↓ SBP	in aorta: ↓ MDA ↑ GSH, Nrf2, HO-1	n/d	in aorta: ↓ pro-caspase-3, cleaved caspase-3, Bnip3	in aorta: ↓ histopathological alterations, contractile response to Phe, autophagy (↓ beclin1, LC3-II) ↑ vasodilatory response to Ach ↔ contractile response to KCl, vasodilatory response to SNP	
SD rats male n/d 200–250 g	CsA-induced (25 mg/kg, 3 weeks, sc.)	REV	75 mg/kg 3 weeks i.g.		↓	↑	↔ SBP, DBP	n/d	in MAs: ↓ p-NFκB P65, p-ERK1/2, p-JNK, p-P38	n/d	↓ TP receptor, contractile response to U46619 ↑ p-AMPKα	[210]
			150 mg/kg 3 weeks i.g.			↑	↓ SBP, DBP	n/d	in MAs: ↓ p-NFκB P65, p-ERK1/2, p-JNK, p-P38	n/d	↓ TP receptor, contractile response to U46619 ↑ p-AMPKα	
C57BL/6 mice male 8 weeks 20 g	salt-sensitive (20–fructose-containing water, 8% NaCl in diet)	PAMK	100 mg/kg 8 weeks orally		↑	n/d	↓ SBP, DBP, MAP ↔ HR	n/d	n/d	n/d	in kidneys: ↓ fibrosis (↓COL-1, COL-3, α-SMA, TGF-β1, β-arrestin 2, ↔ FN, β-arrestin 1), BUN, Scr, glomerulus damage ↔ kidney index	[182]
			300 mg/kg 8 weeks orally			↓ in kidneys	n/d	n/d	in kidneys: ↓ Nlrp3, caspase-1	n/d	in kidneys: ↓ fibrosis (↓COL-1, COL-3, FN, α-SMA, TGF-β1, β-arrestin 1, β-arrestin 2), BUN, Scr, kidney index, glomerulus damage	
C57BL/6 mice pregnant female 10 weeks n/d	pre-eclampsia (AT1-AA 20 μg/g BW in 200 μL of saline, i.v.)	losartan	10 mg/kg GD13-GD19 orally		↓	↑ in kidneys	↓ SBP	in kidneys: ↑ SOD2, FOXO1	n/d	n/d	↓ BW, number of live pups, pup weight, postpartum susceptibility to adriamycin in blood: ↔ glucose in serum: ↔ BUN in urine: ↓ ACR ↔ glucose, sodium in kidneys: ↓ SGLT2 in renal cortex and podocytes, renal injury (↑synaptopodin, nephrin, podocin, p-AMPK)	[173]
		empa	30 mg/kg GD13-GD19 orally			↑ in kidneys	↓ SBP	in kidneys: ↑ SOD2, FOXO1	n/d	n/d	↓ postpartum susceptibility to adriamycin ↔ BW, number of live pups, pup weight in blood: ↔ glucose in serum: ↑ BUN in urine: ↓ ACR ↑ glucose, sodium in kidneys: ↓ SGLT2 in renal cortex and podocytes, renal injury (↑synaptopodin, nephrin, podocin, p-AMPK)	
Wistar pregnant female 10–12 weeks 180–220 g	gestational (L-NAME 250 mg/kg, GD11-GD18, i.p.)	Pae	100 mg/kg GD2-GD19 i.g.		↓	↑ in placenta	↔ MAP	in placenta: ↑ SOD, GSH-Px ↓ MDA	in serum: ↓ TNFα, IL-6, IL-1β, MCP-1, iNOS in placenta: ↓ TNFα, IL-6, IL-1β, MCP-1	n/d	↓ urinary protein in serum: ↑ growth (↑PIGF, VEGF, ↓ sFlt-1), NO in placenta: ↑ growth (↑ PIGF, VEGF, ↓ sFlt-1), eNOS	[176]
			300 mg/kg GD2-GD19 i.g.			↑ in placenta	↓ MAP	in placenta: ↑ SOD, GSH-Px ↓ MDA	in serum: ↓ TNFα, IL-6, IL-1β, MCP-1, iNOS in placenta: ↓ TNFα, IL-6, IL-1β, MCP-1	n/d	↓ urinary protein in serum: ↑ growth (↑PIGF, VEGF, ↓sFlt-1), NO in placenta: ↑ growth (↑PIGF, VEGF, ↓sFlt-1), eNOS	
SD rats female 4–5 months 200–250g	pre-eclampsia (RVPP on GD 14.5)	rSIRT1	constant release by miniosmotic pump GD11.5–GD18.5		↓	↑ in placenta	↓ MAP	n/d	in plasma: ↓ IL-6, TNF-α ↑ NOX	n/d	↑ number of live pups, pup weight in plasma: ↑ angiogenesis (↑VEGF, ↓sFlt-1)	[177]
		rSHH	constant release by miniosmotic pump GD11.5–GD18.5			↑ in placenta	↓ MAP	n/d	in plasma: ↓ IL-6, TNF-α ↑ NOX	n/d	↑ number of live pups, pup weight in plasma: ↑ angiogenesis (↑VEGF, ↓sFlt-1)	
therapeutic												
Wistar rats male 4 weeks 100–120 g	HS-induced (8% NaCl for 8 weeks)	Gen	20 μg/h 4 weeks PVN infusion		↓	↑ in PVN	↓ SBP, DBP, MAP	in PVN: ↑ GSH, SOD, Nrf2, HO-1, Nqo1, T-AOC ↓ MDA, ROS, GSSG, gp91^phox^	in plasma: ↓ TNFα, IL-1β, IL-6 in PVN: ↓ TNFα, IL-1β, IL-6, ac-FOXO1	n/d	↓ HW, cross-sectional area of CMs	[175]
		Gen + NAM	20 μg/h Gen 10 μg/h NAM 4 weeks PVN infusion			↓ in PVN	n/d	in PVN: ↓ GSH, SOD, Nrf2, HO-1, Nqo1, T-AOC ↑ MDA, ROS, GSSG, gp91^phox^	in plasma: ↑ TNFα, IL-1β, IL-6 in PVN: ↑ TNFα, IL-1β, IL-6, ac-FOXO1	n/d	n/d	

Abbreviations: * changes in SIRT1 expression in the model compared with normotensive conditions; 2K1C, two-kidney one-clip; 3-NT, 3-nitrotyrosine; 8-OHdG, 8-hydroxy-2′-deoxyguanosine; ACE, angiotensin converting enzyme; ac-FOXO1, acetylated FOXO1; Ach, acetylcholine; ACR, albumin to creatinine ratio; AKT1, protein kinase B; ALT, alanine transaminase; AMPK, AMP-activated protein kinase; ANP, atrial natriuretic peptide; Apaf-1, apoptotic protease activating factor-1; APP, amyloid precursor protein; As, arsenic; ASC, apoptosis-associated speck-like protein containing a caspase recruitment domain; AST, aspartate aminotransferase; AT1-AA, autoantibodies against angiotensin II type 1 receptor; AT1R, angiotensin II type 1 receptor; BAIBA, beta aminoisobutyric acid; Bax, Bcl2–associated X protein; Bcl2, B-cell lymphoma 2; BCL2L1, Bcl2 Like 1; Bcl-XL, B-cell lymphoma extra-large; Bnip3, Bcl-2 interacting protein 3; BNP, brain natriuretic peptide; BP, blood pressure; BUN, blood urea nitrogen; BW, body weight; CCND1, cyclin D1; CK, creatine kinase; COL1A1, collagen type I α 1; CTGF, connective tissue growth factor; CTNNB1, catenin beta 1; cyto, cytochrome; DBP, diastolic blood pressure; EF, ejection fraction; EGCG, epigallocatechin-3-gallate; EndoG, endonuclease G; eNOS, endothelial nitric oxide synthase; empa, empagliflozin; EPI, epinephrine; Fas, type I integral membrane protein; Fas L, Fas ligand; FADD, Fas-associated protein with death domain; FGF21, fibroblast growth factor; FN, fibronectin; FOXO, forkhead box O; FS, fractional shortening; GAD67, 67 kDa glutamic acid decarboxylase; GD, gestational day; Gen, genistein; Giα-2, guanine nucleotide regulatory protein; gp91phox, glycoprotein of 91 kDa phagocyte oxidase; GSH, glutathione; GSH-Px, glutathione peroxidase; GSSG, glutathione disulfide; H3K9ac, histone H3 lysine 9 acetylation; HDAC1, histone deacetylase 1; HDL, high-density lipoprotein; HFD, high fat diet; HSP90AA1, heat shock protein 90 alpha family class A member 1; HSP90AB1, heat shock protein 90 alpha family class B member 1; HTN, hypertension; HO-1, heme oxygenase 1; HR, heart rate, HW, heart weight; IGF1, insulin-like growth factor 1; IGF-1R, IGF-1 receptor; IGF-II, insulin-like growth factor II; IL, interleukin; IκBα, inhibitor of NF-κB; iNOS, inducible nitric oxide synthase; IVSd, interventricular septum at diastole; KCl, potassium chloride; LC3-II, microtubule-associated protein 1A/1B-light chain 3-phosphatidylethanolamine conjugate; LDH, lactate dehydrogenase; LDL-C, low-density lipoprotein cholesterol; LV, left ventricle; LVEF, left ventricular ejection fraction; LVIDd, internal dimension at diastole of left ventricle; LVIDs, internal dimension at the systole of the left ventricle; LVMI, left ventricular mass index; LVPWd, left ventricular posterior wall thickness at diastole; LVW, left ventricular weight; MAP, mean arterial pressure; MAPK, mitogen-activated protein kinase; MAs, mesenteric arteries; MCP-1, monocyte chemoattractant protein 1; MDA, malondialdehyde; Mel, melatonin; MT, media thickness; MT/LD, media thickness to lumen diameter ratio; mtDNA, mitochondrial DNA; mTOR, mechanistic target of rapamycin; MV E/A, mitral valve early diastolic to late diastolic wave velocity ratio; MYH7, myosin heavy chain 7; NADPH, reduced nicotinamide adenine dinucleotide phosphate; NAFTC3, nuclear factor of activated T cells 3; NAM, nicotinamide; NE, norepinephrine; NF-κB, nuclear factor kappa B; NRF1, nuclear respiratory factor 1; Nlrp3, pyrin domain-containing protein 3; NO, nitric oxide; NOX, NADPH oxidase subunit; Nqo1, NAD(P)H:quinone oxidoreductase; n-tyr, tyrosine nitration, ONOO^−^, peroxynitrite; OPN, osteopontin; p21, cyclin-depended kinase inhibitor protein; p-P38, phosphorylated p38 mitogen-activated protein kinase; p47phox, cytosolic subunit of the NADPH oxidase; p55, palmitoylated erythrocyte membrane protein; P65, activated B cells protein; p-PI3K, phosphorylated Phosphoinositide 3-kinase; Pae, paeoniflorin; PAMK, polysaccharide of Atractylodes macrocephala Koidz; p-AMPK, phosphorylated AMPK; PCNA, proliferating cell nuclear antigen; Phe, phenylephrine; p-eNOS, phosphorylated eNOS; p-ERK1/2, phosphorylated extracellular signal-regulated kinase 1/2; p-FOXO3α, phosphorylated FOXO isoform; pGATA4, phosphorylated GATA binding protein 4; PGC-1α, activated receptor gamma coactivator 1α; PGQYD, Pinggan-Qianyang decoction; PIGF, placental growth factor; PIK3CA, phosphatidylinositol-4,5-bisphosphate 3-kinase catalytic subunit alpha; p-IkBα, phosphorylated IκB kinase alpha; p-IKKβ, phosphorylated IκB kinase beta; p-JNK, phosphorylated c-Jun N-terminal kinase; PP, pulse pressure; p-P65, phosphorylated P65; PPARG, peroxisome proliferator-activated receptor gamma; PRKCA, protein kinase C alpha; PTEN, phosphatase and tensin homology detected on chromosome ten; PTGS2, prostaglandin-endoperoxide synthase; PVN, paraventricular nucleus; QYYYG, Qianyang Yuyin granule; REV, resveratrol; ROS, reactive oxygen species; RSNA, renal sympathetic nerve activity; rSHH, recombinant Sonic Hedgehog protein; RVLM, rostral ventrolateral medulla; SA-β-gal, senescence-associated β-galactosidase; SBP, systolic blood pressure; SCR, serum creatinine; sFlt-1, soluble fms-like tyrosine kinase-1; SGLT2, sodium-glucose cotransporter 2; SHR, spontaneously hypertensive rats; shRNA, short hairpin RNA; SIRT3, sirtuin 3; SOD, superoxide dismutase; T-AOC, total antioxidant capacity; t-Bid, truncated BH3-interacting domain death agonist; T-CHO, total cholesterol; TFAM, mitochondrial transcription factor A; TFB2M, mitochondrial transcription factor B2; TGF-β1, tissue growth factor β1; TH, tyrosine hydroxylase; TLR4, tool-like receptor 4; TNFα,tumor necrosis factor alpha; TP receptor, TxA2/prostanoid receptor; uPA, urokinase-type plasminogen activator protein; VEGF, vascular endothelial growth factor; VH4, peptide composed of Valine-Histidine-Valine-Valine; VSMCs, vascular smooth muscle cells; WT, weight; α-SMA, alpha smooth muscle actin; α-MG, alpha-mangostin.

The available evidence does not establish a unidirectional relationship between SIRT1 and hypertension. Rather, SIRT1 alterations may represent both a response to hypertension-associated stimuli and a contributing factor to hypertension, depending on the tissue, experimental model and direction of SIRT1 modulation. The findings point to SIRT1 as a context-dependent regulator of cellular homeostasis, integrating metabolic, mitochondrial, redox, inflammatory, and vascular signaling pathways, rather than a uniformly protective or detrimental factor.

**Table 3:** The most studied SIRT1 targets in hypertension in in vivo studies.

Target	Target Function	Effect of SIRT1 Activation	Tissues	Ref.
NOX	pro-oxidative	↓	mesenteric arteries, PVN	[30,171,174]
SOD	anti-oxidative	↑	aorta, mesenteric arteries, PVN, kidneys	[30,136,171,178]
IL-1β	anti-oxidative	↑	mesenteric arteries, PVN	[171,174,175]
IL-6	anti-oxidative	↑	PVN, heart	[84,174,175]
TNF-α	pro-inflammatory	↓	PVN, heart	[84,174,175]
Bcl2	anti-apoptotic	↑	heart, cerebral cortex	[83,149,169,178]
Bcl-xl	anti-apoptotic	↑	heart, cerebral cortex	[83,149,169,178]
Akt	anti-apoptotic	↑	heart, cerebral cortex	[83,84,149,169]
PI3K	anti-apoptotic	↑	heart, cerebral cortex	[83,84,149,169]
COL1A1	profibrotic	↓	heart, kidneys	[84,169,172,182]

Abbreviations: Akt, protein kinase B; Bcl2, B-cell lymphoma 2; Bcl-xl, B-cell lymphoma extra-large; COL1A1, collagen type I α 1; IL-1β, interleukin 1β; IL-6, interleukin 6; NF-κB, nuclear factor kappa B; NOX, NADPH oxidase subunit; PI3K, phosphoinositide 3-kinase; SOD, superoxide dismutase; TNFα, tumor necrosis factor α.

**Table 4:** Effects of SIRT1 activation on hypertension: evidence from in vitro studies.

Material	Model Characteristics	Modulator		SIRT1 Expression in Model			Effects					Ref.
							SIRT1	Oxidative Stress	Inflammation	Apoptosis	Others	
human cells												
HUVECs	K.pn. EVs 50 ng/mL 24/48 h	REV 20 μM		24 h	50 ng/mL	↓	n/d	n/d	n/d	n/d	↓ senescence (↓ SA β-gal-positive cells (48 h incubation))	[137]
					5 ng/mL							
					0.5 ng/mL							
		SIRT1 OE, MOI 100 co-incubation with K.pn. EVs for 24/48 h		48 h	50 ng/mL	↓	↑	↓ NOX2	n/d	n/d	↑ endothelial function (↑ p-eNOS, NO, ↓ COX-2) ↓ senescence (↓ p53)	
					5 ng/mL	↔						
					0.5 ng/mL	↔						
HUVECs	Ang II 1 μmol/L 24 h	si-PDE4B for 48 h, after Ang II stimulation		↓			↑	↑ Nrf2, HO-1, NQO1	n/d	↓ CHOP, caspase 12, caspase 3	↑ p-AMPK, endothelial function (↑ NO, p-eNOS, ↔ eNOS, ↓ ET-1), migration, angiogenesis ↔ AMPK ↓ endoplasmic reticulum stress (↓ GRP78, ATF6, p-PERK, ↔ PERK), mitochondrial damage (↑ mtDNA copy)	[211]
HUVECs	Ang II 1 μM 24 h	bazedoxifene 24 h, 1 h prior to Ang II induction	2 μM	↓			↑	↑ NO, p-eNOS ↓ ROS ↔ P47^phox^	n/d	↓ in assay, Bax, cleaved caspase-3 ↔ Bcl-2	↔ viability	[141]
			4 μM				↑	↑ NO ↓ ROS, P47^phox^ ↔ p-eNOS	n/d	↑ Bcl-2 ↓ in assay, Bax, cleaved caspase-3	↑ viability	
			6 μM				↑	↑ NO, p-eNOS ↓ ROS, P47^phox^	n/d	↑ Bcl-2 ↓ in assay, Bax, cleaved caspase-3	↑ viability	
		bazedoxifene 24 h, 1 h prior to Ang II induction + si-SIRT1	4 μM				↓	↓ NO ↑ ROS, p47^phox^ ↔ p-eNOS	n/d	↑ in assay, Bax, cleaved caspase-3 ↔ Bcl-2	n/d	
healthy pregnant women HUVECs	incubated in 5% CO_2_	miR-199a-5p-OE exosomes derived from sPE, 40 μg/mL		n/d			n/d	↑ ROS	n/d	n/d	↓ endothelial function (↓ NO, ↑ ICAM-1, VCAM-1), proliferation ↑ wound healing rate	[212]
		EX527 10 μM, 24 h					n/d	↑ ROS	n/d	n/d	↓ endothelial function (↓ NO, ↑ ICAM-1, VCAM-1)	
HUVECs	incubated with plasma from GH patients, 24 h	trans-resveratrol, 10 μM, 24 h					n/d	↓ ROS ↑ antioxidant activity	n/d	n/d	↔ NO, nitrate	[193]
		trans-resveratrol, 10 μM + EX527 30 μM, 24 h					n/d	↑ ROS ↓ antioxidant activity	n/d	n/d	↔ NO, nitrate	
	incubated with plasma from PE patients, 24 h	trans-resveratrol, 10 μM, 24 h					n/d	↔ ROS, antioxidant activity	n/d	n/d	↔ NO, nitrate	
		trans-resveratrol, 10 μM + EX527 30 μM, 24 h					n/d	↑ ROS, antioxidant activity	n/d	n/d	↑ NO, nitrate	
HPVECs	H/R-induced (hypoxia 5% CO_2_, 95% N2 for 4 h, normoxia 5% CO_2_, 95% air for 6 h)	GDF-15 100 ng/mL		↓			↑	n/d	n/d	↑ p-AMPK ↓ in assay	n/d	[183]
		GDF-15 100 ng/mL, compound C					↓	n/d	n/d	↓ p-AMPK ↑ in assay		
isolated vessels												
mouse aorta	K.pn. EVs 50 ng/mL 24/48 h	REV 20 μM		n/d			n/d	↓ NADPH oxidase	n/d	n/d	↑ relaxation in response to Ach	[137]
		SIRT1 OE, MOI 100 co-incubation with K.pn. EVs for 24 h										
aorta of 10 weeks C57BL/6 male mouse	*Klebsiella Pneumoniae*-induced HTN (10^9^ CFU *K.pn.* once every 2 days for 4 weeks, by gavage)	REV 20 μM 24 h incubation					n/d	↓ NADPH oxidase, superoxide in ECs	n/d	n/d	↑ relaxation in response to Ach	
aortas of 10–12 weeks, 280–310 g male SHR	SHR SBP ≥ 150 mmHg	EMPA 10 μM, EX527 1 μM		↑			n/d	n/d	n/d	n/d	↓ potency of Ach-mediated relaxation ↔ maximum relaxation response to Ach	[181]
MAs of 10–12 weeks, 280–310 g male SHR		EMPA 10 μM, EX527 1 μM					n/d	n/d	n/d	n/d	↑ potency of U46619-mediated constriction ↓ maximum relaxation response to Ach ↔ potency of Ach-mediated relaxation, potency of Phe-mediated constriction, maximum contractile response to Phe, maximum contractile response to U46619	
jejunal and ileal arteries branch of MAs of 200–250g male SD rats with CsA-induced HTN	CsA 10 μM 24 h	REV 10 or 100 μM, 24 h during incubation with CsA		↓			↑	n/d	n/d	n/d	↓ TP receptor, contractile response to U46619	[210]
cardiac cells												
HL-1 cells	HS-stimulated (NaCl 100 mM)	SIRT1 OE 2 μg 48 h before HS stimulation		↓			↑	↑ Nrf2, NQO1, HO-1, GSH ↓ ROS, MDA, SOD	n/d	↓ in assay	↑ proliferation ↓ ferroptosis (↓ PTGS2, 4HNE, FTH1, Fe^2+^, ↑ GPX4)	[186]
rat H9c2 cells	Ang II 1 μM 24 h	QYYYG (+ Ang II) 24 h		50 μg/mL			↓	↔ expression ↑ mRNA	n/d	n/d	n/d	[170]
				100 μg/mL				↑ expression and mRNA	n/d	n/d	n/d	
rat H9c2 cells	NaCl 150 μmol/L	BMTN 5 μmol/L, 8 h		↓			↑	n/d	n/d	↓ Bax, p-p38, in assay ↔ Bcl-2, p-AMPK	↓ ATP	[185]
		geniposide 0.1 μmol/L, 8 h					↑	n/d	n/d	↓ Bax, p-p38, in assay ↔ Bcl-2, p-AMPK	↔ viability, ATP	
		geniposide 0.5 μmol/L, 8 h					↑	n/d	n/d	↑ Bcl-2, p-AMPK ↓ Bax, p-p38	↑ viability ↔ ATP	
		geniposide 5 μmol/L, 8 h					↑	n/d	n/d	↑ Bcl-2, p-AMPK ↓ Bax, p-p38	↑ viability ↓ ATP	
endothelial cells												
mouse aortic ECs	K.pn. EVs 50 ng/mL 24/48 h	SIRT1 OE, MOI 100 co-incubation with K.pn. EVs for 24 h		n/d			n/d	↓ superoxide anion level	n/d	n/d	n/d	[137]
muscle cells												
aortic VSMCs	SHR	losartan 10^−5^ mol/L, 24 h		↑			↓	n/d	n/d	n/d	n/d	[140]
		NAM 5 mmol/L, 24 h					n/d	↓ H_2_O_2_	n/d	n/d	↓ Giα-2, Giα-3	
		EX527 5 × 10^−5^ mol/L					n/d	n/d	n/d	n/d	↓ Giα-2, Giα-3	
		siSIRT1 48 h					n/d	↓ NOX2, P47^phox^	n/d	n/d	↓ Giα-2, Giα-3, DNA synthesis, cell cycle regulatory proteins (↓ cyclin D1, Cdk4, p-Rb), proliferation (↓ p-EGF-R, p-ERK1/2, p-Akt)	
kidney cells												
HK2 cells	Ang II 0.1 nM 12 h	PAMK 50 μg/mL		↑			↓	n/d	↓ TNF-α, Nlrp3, caspase-1, IL-1β, IL-18	n/d	↓ fibrosis (↓ COL-1, FN, α-SMA)	[182]
		PAMK 50 μg/mL, 12 h + siSIRT1					↔	n/d	↓ TNF-α, IL-1β ↔ Nlrp3, caspase-1, IL-18	n/d	n/d	
HK2 cells	oleic acid 200 μM	IBMX 4 μM		↓			↑	n/d	n/d	n/d	↑ cAMP, PKA, EPAC1, p-PKA ↓ p300, ACE1, H3K27ac	[184]
NRK-52E cells	Ang II 10^−7^ mM (0.1 nM)	REV 25 μM		↓			↑	↑ SOD ↓ MDA	n/d	n/d	↓ fibrosis (↓ Col-1, α-SMA), autophagy (↓ LC3II, beclin-1, ↑ FOXO3a)	[172]
		*C. cicadae* 10%					↑	↑ SOD ↓ MDA	n/d	n/d	↓ fibrosis (↓ Col-1, α-SMA), autophagy (↓ LC3II, p62, beclin-1, ↑ FOXO3a)	
placenta												
HTR-8/SVneo cells	incubated in 5% CO_2_	AIF 2 μM, 48 h		n/d			↔	n/d	n/d	n/d	↔ proliferation-related signaling (↔ p-Akt, p-ERK1/2, p-P38, p-AMPK)	[51]
		AIF 5 μM, 48 h					↑	n/d	n/d	n/d	proliferation-related signaling (↑ p-ERK1/2, p-AMPK, ↔ p-Akt, p-P38)	
		AIF 10 μM, 48 h					↑	↑ SOD2, SOD3, CAT	n/d	↓ late apoptotic cells	↑ proliferation-related signaling (↑ p-Akt, p-ERK1/2, p-P38, p-AMPK), migration (↓ area between cells population, ↑ invasive cells, MMP13, MMP14), mitochondrial function (↓ loss of mitochondrial membrane potential, ↑ respiration, ATP, ETC genes)	
	H_2_O_2_ 200 μM	AIF 2, 5, 10 μM, 30 min (ROS) or 48 h (proliferation, mitochondrial membrane potential)					n/d	↓ ROS	n/d	↓ late apoptotic cells	↑ proliferation, loss of mitochondrial membrane potential	
	siSIRT1 20 nM, 24 h before AIF	AIF 2, 5, 10 μM, 48 h						n/d	n/d	n/d	↓ proliferation	
HTR-8/SVneo cells	miR-494 75 nM	SIRT1 plasmids 1.17 μg		↓			↑	↓ ROS	↓ NLRP3, IL-1β	n/d	↑ migration, proliferation, ATP, G0/G1 to S phase progression ↓ aging (↓ SaβG, P21, ↔ P16)	[187]
adrenal medulla												
rat PC12 cells	Ang II 0.1 μM	REV 25 μM		↓			↑	↑ SOD1, FOXO1	n/d	n/d	n/d	[150]

Abbreviations: 4HNE, 4-hydroxy-2-nonenal; ACE1, angiotensin converting enzyme 1; Ach, acetylcholine; ac-NF-κB P65, acetylated NF-κB P65 subunit; ac-STAT3, acetylated STAT3; AIF, alpinumisoflavone; AMPK, AMP-activated protein kinase; Ang II, angiotensin II; ATF6, activating transcription factor 6; ATP, adenosine triphosphate; Bax, B-cell lymphoma 2-associated X protein; Bcl2, B-cell lymphoma 2; BAIBA, β-aminoisobutyric acid; BMTN, bumetanide; cAMP, 3′,5′-cyclic adenosine monophosphate; Cdk4, cyclin-dependent kinase 4; CHOP, C/EBP homologous protein; COL-1, collagen type I; COX-2, cyclo-oxygenase 2; CsA, cyclosporine A; ECs, endothelial cells; EMPA, empagliflozin; eNOS, endothelial nitric oxide synthase; EPAC1, cAMP dependent exchange protein 1; ET-1, endothelin 1; Fe^2+^, iron cations; FN, fibronectin; FOXO1, forkhead box O isoform; FTH1, ferritin heavy chain 1; Gclc, glutamate-cystine ligase catalytic subunit; Gclm, cystine ligase modifier subunit; GDF-15, growth differentiation factor 15; GH, gestational hypertension; GPX4, glutathione peroxidase 4; Giα, inhibitory guanine nucleotide regulatory protein α; GRP78, glucose related protein 78; GSH, glutathione; H3K27ac, histone 3 lysine 27 acetylation; HO-1, heme oxygenase 1, HPVECs, human placental vascular endothelial cells; HS, high salt; HTN, hypertension; HUVECs, human umbilical venous endothelial cells; IBMX, 3-isobutyl-1-methylxanthine; ICAM-1, intercellular adhesion molecule 1; IL, interleukin; K.pn. EVs, Klebsiella pneumoniae-secreted extracellular vesicles; Keap1, Kelch-like ECH-associated protein 1; MAs; mesenteric arteries; MCP-1, monocyte chemoattractant protein; MDA, malondialdehyde; MMP, matrix metalloproteinase; MOI, multiplicity of infection; m-p47phox, methylated cytosolic subunit of the NADPH oxidase; mtDNA, mitochondrial DNA; MYH7, Myosin heavy chain 7; NADPH, reduced nicotinamide adenine dinucleotide phosphate; NAM, nicotinamide; NF-κB P65, nuclear factor kappa B P65 subunit; Nlrp3, pyrin domain-containing protein 3; NO, nitric oxide; NOX, NADPH oxidase subunit; NPPA, natriuretic peptide precursor A; NPPB, natriuretic peptide precursor B; NQO1, NAD(P)H:quinone oxidoreductase 1; NRF1, nuclear respiratory factor 1; Nrf2, nuclear factor erythroid 2-related factor 2; OE, overexpression; Opn, osteopontin; p-IkBα, phosphorylated IκB kinase beta; p300, E1A-associated 300 kDa protein; p47phox, cytosolic subunit of the NADPH oxidase; p53, tumor protein; p-Akt, phosphorylated protein kinase B; PAMK, polysaccharide of Atractylodes macrocephala Koidz; p-AMPK, phosphorylated AMPK; PCNA, proliferating cell nuclear antigen; p-EGF-R, phosphorylated epidermal growth factor receptor; p-eNOS, phosphorylated eNOS; PERK, protein kinase RNA-activated-like endoplasmic reticulum kinase; p-ERK1/2, phosphorylated extracellular signal-regulated kinase 1/2; PGC-1α, activated receptor gamma coactivator 1α; PE, phenylephrine; PKA, protein kinase A; p-NFκB P65, phosphorylated NF-κB P65 subunit; p-p38, phosphorylated p38 mitogen-activated protein kinase; p-p47phox, phosphorylated cytosolic subunit of the NADPH oxidase; p-PERK, phosphorylated PERK; p-PKA, phosphorylated PKA; p-Rb, phosphorylated retinoblastoma protein; p-STAT3, phosphorylated STAT3; PTGS2, prostaglandin-endoperoxide synthase; QYYYG, Qianyang Yuyin granule; REV, resveratrol; ROS, reactive oxygen species; SA-β-gal, senescence-associated β-galactosidase; SBP, systolic blood pressure; SHR, spontaneously hypertensive rat; si-PDE4B, phosphodiesterase 4B small interfering RNA; siSIRT1, small interfering SIRT1 RNA; SM22α, smooth muscle 22α; SOD, superoxide dismutase; sPE, severe preeclampsia; TGF-β1, tissue growth factor β1; t-IkBα, total IκB kinase alpha; TNF-α, tumor necrosis factor alpha; TP receptor, TxA2/prostanoid receptor; U46619, synthetic thromboxane A2; VCAM-1, vascular cell adhesion molecule; VSMCs, vascular smooth muscle cells; α-SMA, alpha smooth muscle actin.

**Table 5:** The most studied SIRT1 targets in hypertension in in vitro studies.

Target	Target Function	Effect of SIRT1 Activation	Cells	Ref.
NOX	pro-oxidative	↓	HUVECs	[137,140]
Nrf2	anti-oxidative	↑	HUVECs, HL-1	[183,186]
HO-1	anti-oxidative	↑	HUVECs, HL-1	[183,186]
Nqo1	anti-oxidative	↑	HUVECs, HL-1	[183,186]
SOD	anti-oxidative	↑	PC12, NRK-52E, HTR-8/SVneo	[51,150,172]
Bax	pro-apoptotic	↓	HUVECs, H9c2	[141,185]
Bcl2	anti-apoptotic	↑	HUVECs, H9c2	[141,185]
AMPK	anti-apoptotic	↑	HUVECs, H9c2	[183,185]

Abbreviations: AMPK, AMP-activated protein kinase; Bax, B-cell lymphoma 2-associated X protein; Bcl2, B-cell lymphoma 2; HO-1, heme oxygenase 1; NOX, NADPH oxidase subunit; Nqo1, NAD(P)H:quinone oxidoreductase 1; Nrf2, nuclear factor erythroid 2-related factor 2; SOD, superoxide dismutase; TNFα, tumor necrosis factor alpha.

### 4.3. Effects in Humans

In this review, we analyzed both observational studies and clinical trials investigating the role of SIRT1 in hypertension in humans (Table 6 and Table 7). Only two studies involved patients with primary hypertension, whereas the remaining studies focused on hypertension during pregnancy. These studies consistently reported reduced SIRT1 levels in hypertensive patients. In a cohort study of young individuals, SIRT1 levels were negatively correlated with BP in males but not in females, highlighting the importance of considering sex-specific differences in future investigations of SIRT1 in hypertension [184]. Additionally, SIRT1 showed a positive correlation with MAP within the normal range, but a negative correlation at higher MAP values [213], as well as with SBP and DBP [214]. Studies in women with PE indicated that SIRT1 may have diagnostic value for disease severity, as patients with more severe PE exhibited markedly lower SIRT1 levels [214]. Furthermore, SIRT1 was consistently downregulated in both blood and placental tissue in PE [187,214,215,216,217,218].

More specifically, among the two observational studies conducted in primary hypertension, Wang et al. [184] demonstrated decreased serum SIRT1 levels in children and adolescents with hypertension. Importantly, the authors showed a significant inverse association between SIRT1 and BP only in males, whereas no such relationship was observed in females. Moreover, lower SIRT1 concentrations were found in patients with left ventricular hypertrophy, and SIRT1 was negatively correlated with left ventricular mass Z-score, suggesting that reduced SIRT1 may be linked not only to BP elevation itself but also to early hypertension-mediated cardiac remodeling. Similarly, Yeganeh-Hajahmadi et al. [219] reported lower SIRT1 levels in hypertensive adults, with an even more pronounced reduction in patients with concomitant diabetes and hypertension. Their findings are particularly relevant from a pathophysiological perspective, because SIRT1 correlated positively with MAP within the physiological range but negatively at higher MAP values, which may indicate that the relationship between SIRT1 and BP is not linear and changes with disease progression. In addition, the positive association between SIRT1 and Klotho supports the concept that both proteins are linked to vascular aging and endothelial protection.

The studies focused on hypertension in pregnancy yielded slightly more heterogeneous but still clinically meaningful results. In pregnancy-induced hypertension, Chen and Shi [183] observed decreased serum SIRT1 levels, supporting the concept that impaired SIRT1 signaling may already be present in hypertensive complications of pregnancy. In contrast, Golańska-Wróblewska et al. [220] did not find significant differences in plasma SIRT1 in GH, suggesting that isolated GH may differ from PE with respect to the degree of endothelial and placental involvement. This discrepancy may reflect differences in disease severity, timing of sampling, biological material analyzed or the underlying pathomechanisms of the hypertensive disorder.

The most consistent findings concerned PE. Pei et al. [221] showed reduced SIRT1 levels in women with PE and additionally demonstrated decreased placental SIRT1 expression in placentas obtained from women with severe features of the disease, indicating that systemic and placental SIRT1 downregulation coexist in clinically advanced PE. Broady et al. [215] also reported decreased SIRT1 in pregnant women with PE, reinforcing the consistency of this observation across independent cohorts. Ge and Kong [214] further refined the clinical significance of SIRT1 by showing that patients with severe PE had lower SIRT1 levels than those with mild PE. Moreover, SIRT1 was negatively correlated with SBP, DBP and random urinary protein levels, which suggests that lower SIRT1 reflects both hemodynamic severity and the extent of renal involvement. Importantly, the authors also pointed to the high diagnostic value of SIRT1 for assessing PE severity, making it one of the most clinically informative studies in the table. Yu et al. [216] confirmed decreased circulating SIRT1 in severe PE and additionally showed lower placental SIRT1 expression. Their study also linked SIRT1 to established angiogenic markers: SIRT1 was positively correlated with PlGF and negatively correlated with sFlt-1, which strongly supports its relationship with placental vascular dysfunction. Su et al. [187] reported reduced placental SIRT1 expression in severe PE, further supporting the hypothesis that SIRT1 deficiency is a local placental feature of the disorder. Likewise, Wang et al. [217] found decreased placental SIRT1 in women with PE associated with premature placental aging and grade III placenta before 37 weeks of gestation, suggesting that SIRT1 downregulation may also be related to accelerated placental senescence. Finally, Viana-Mattioli et al. [218] demonstrated lower plasma SIRT1 in PE than in gestational hypertension, indicating that SIRT1 may help discriminate between more severe endothelial-placental disease and less complex hypertensive states during pregnancy.

Taken together, these studies indicate that low SIRT1 is associated with both primary hypertension and hypertensive disorders of pregnancy, although the strength and consistency of this association appear greater in pre-eclampsia than in gestational hypertension. In primary hypertension, reduced SIRT1 seems to be linked with BP values, sex-related differences, left ventricular hypertrophy, diabetes burden, and Klotho-related vascular aging. In pregnancy, the most robust data support a relationship between low SIRT1 and PE severity, placental dysfunction, angiogenic imbalance, and premature placental aging.

In the completed randomized crossover double-blind trial by Marques et al. [222], [NCT02616822], a single 300 mg dose of trans-resveratrol administered to treated hypertensive patients with endothelial dysfunction improved flow-mediated dilation (FMD). This finding is clinically important because it suggests that SIRT1-activating compounds may exert beneficial vascular effects even in the absence of an acute hypotensive response. Thus, endothelial improvement may precede measurable BP reduction or may occur independently of it.

The ongoing open-label randomized study NCT06828211 is evaluating trans-resveratrol 500 mg daily for 3 months in postmenopausal women with stage I-II hypertension and reduced bone mineral density. Although results are not yet available, the design of this trial is noteworthy because it addresses a population at increased cardiometabolic and vascular risk, in whom SIRT1 activation could theoretically affect not only BP control but also endothelial function, cardiac remodeling, and age-related vascular decline. Therefore, this trial may provide valuable information on the longer-term efficacy and tolerability of REV in a clinically relevant hypertensive population.

In the FLAVO trial, Dower et al. [213,223]; [NCT01691404] investigated quercetin-3-glucoside supplementation in untreated prehypertensive and hypertensive individuals. In this study, IL-1β levels decreased, indicating an anti-inflammatory effect. Since SIRT1 is closely linked with suppression of inflammatory signaling, these findings suggest that some clinical benefits of SIRT1-related interventions may be mediated through attenuation of low-grade inflammation rather than through direct short-term BP lowering.

Finally, the phase 4 randomized double-blind trial NCT04790942 evaluating berberine hydrochloride in patients with primary hypertension remains of particular interest, because berberine is one of the better-known SIRT1-activating compounds with pleiotropic metabolic and vascular effects. However, as the study status is listed as unknown and outcome data are not yet available, its contribution is currently limited to demonstrating growing clinical interest in SIRT1-oriented therapeutic approaches rather than providing definitive efficacy evidence.

Overall, the clinical trials suggest that interventions linked to SIRT1 activation may improve endothelial function and inflammatory status even when BP remains unchanged. This is consistent with the broader mechanistic role of SIRT1 in vascular protection, antioxidative defense and endothelial homeostasis. At present, however, the number of completed human trials is small, the study populations are heterogeneous, and most available interventions are indirect SIRT1 modulators rather than selective pharmacological activators. Consequently, although current data support the biological and clinical relevance of SIRT1 in hypertension, larger prospective studies and adequately powered randomized controlled trials are still needed to determine whether SIRT1 can be implemented as a reliable biomarker or therapeutic target in routine clinical practice.

Essential hypertension is often considered and managed as a homogeneous condition. Nevertheless, it can be phenotypically classified based on underlying hemodynamic patterns beyond systolic and diastolic values. Arterial BP consists of a steady component: MAP determined by cardiac output (CO) and peripheral vascular resistance (PVR), as well as a pulsatile component (pulse pressure—PP) primarily influenced by stroke volume (SV) and arterial stiffness [224]. Phenotyping according to age and sex may facilitate a more tailored therapeutic approach based on the predominant hemodynamic mechanisms underlying the disease in specific patient groups [225].

In young adults characterised by high elasticity of the vascular tree, isolated systolic hypertension (ISH) is primarily driven by increased CO and SV [226], whereas in individuals with systolic–diastolic hypertension (SDH) increased PVR is the predominant underlying mechanism [227]. It has been postulated that the contribution of PVR to hypertension in seniors is also essential and underestimated, given that vascular resistance continues to rise with age [228].

PVR is governed by small vessels of <300 μm in diameter and represents a key hemodynamic feature of essential hypertension [229]. Microvascular resistance is elevated through augmented vasoconstriction, structural remodelling of pre-capillary vessels, and rarefaction of arterioles and capillaries, ultimately leading to HMOD [230]. Microvascular abnormalities may both result from and exacerbate hypertension, creating a vicious cycle in which increased microvascular resistance further elevates BP [229]. Patients with hypertension exhibit impaired endothelium-dependent vasodilation, and data from the Framingham Heart Study indicate that the extent of endothelial dysfunction correlates positively with hypertension severity [10,231].

Patients with Bartter’s or Gitelman’s syndromes (BS/GS), who typically present with normo- or hypotension, upregulated NO signalling, enhanced endothelium-dependent vasodilation, and reduced oxidative stress, were used as a model to investigate the relationship between SIRT1 and NO levels, compared with healthy and hypertensive individuals [232]. A significant correlation between SIRT1 and flow-mediated dilation (FMD), assessed by B-mode scan of the right brachial artery was observed only in BS/GS patients. The association of elevated SIRT1 with increased FMD in this group reinforces the link between SIRT1, NO, and vascular function in humans suggesting that reduced SIRT1 may contribute to endothelial dysfunction in hypertension. This concept is further supported by recent human data demonstrating that hypertension is associated with reduced SIRT1 gene expression, accompanied by impaired antioxidant defenses and enhanced oxidative stress, which may accelerate vascular aging and endothelial injury [233]. Therefore, reduced SIRT1 may represent not only a mechanistic contributor but also a clinically relevant biomarker of vascular dysfunction in hypertensive patients.

In the Framingham Heart Study, 37% of subjects had hypertension. SBP increased linearly from 30 to 84 years of age, accompanied by a parallel rise in DBP. However, after the age of 50–60 years, DBP began to decline. This late decrease in DBP, occurring alongside a continued rise in SBP, is consistent with increasing large artery stiffness [228]. Therefore, ISH becomes more frequent with age, whereas the percentage of patients with isolated diastolic hypertension (IDH) declines over time. The middle-aged patients predominantly suffer from SDH. In older adults, isolated systolic hypertension predominates and drives CV risk, so its management largely parallels that of combined systolic–diastolic hypertension in younger individuals [2]. Although initially lower than in men, women’s BP rises to comparable levels by the sixth decade and often slightly exceeds men’s thereafter [228]. Because SIRT1 participates in eNOS activation, mitochondrial homeostasis, and antioxidative signaling, age-related SIRT1 decline may be one of the mechanisms linking vascular aging with progressive systolic hypertension and widened pulse pressure.

In a study of the Anglo-Cardiff Collaborative Trial population, the overall prevalence of hypertension was 27.6% (ISH = 14.5%, SDH = 9.9%, IDH = 3.2%) [234]. Considering sex differences, in young participants, ISH was most common in men, whereas in women, SDH and IDH predominated. Between 30 and 60 years, SDH was the most prevalent form of hypertension higher body mass index (BMI) was observed in men across all hypertension phenotypes n both sexes, while in individuals over 60 years, ISH became the most common, with no significant sex difference in prevalence. Compared with normotensive individuals, under 30 and over 60 years, as well as in middle-aged men with SDH or IDH. In women, higher BMI was observed under 30 years in those with SDH or IDH, over 60 years in those with ISH, and in middle-aged women across all hypertension phenotype [225]. These observations may be clinically relevant because SIRT1 is linked to adipose tissue biology, insulin sensitivity, inflammatory tone, and metabolic flexibility; thus, reduced SIRT1 activity may contribute to obesity-associated hypertension phenotypes and sex-specific cardiometabolic risk profiles.

In a cohort study of the UK Biobank, three hypertension phenotypes with distinct risk profiles, CV imaging features, and outcomes were identified despite relatively minor differences in BP [234]. The incidence of major adverse cardiovascular event (MACE) was more frequently in a cluster characterised by LV hypertrophy, geometric remodelling (mass-to-volume ratio), impaired LV mechanics (global longitudinal strain) but preserved EF, altered passive left atrial emptying fraction, LV EF, and LV volumes assessed by cardiac magnetic resonance (CMR). In this patient group, men predominated, comprising 99.9% of participants. The most frequent MACEs in described group apart from atherosclerotic events were HF and atrial fibrillation. These results highlight LV hypertrophy as a prognostic marker in hypertensive heart disease [235].

Most of the hypertension-related morphological changes and some of the following clinical complications are linked to remodelling. Cardiac hypertrophy is the heart’s primary response to chronic hemodynamic preload and/or overload to preserve CO [236]. Chronic LV pressure overload primarily induces wall thickening and concentric hypertrophy, whereas chronic LV volume overload leads to chamber enlargement and eccentric hypertrophy [237]. Initially, hypertrophic growth reduces wall stress and preserves function, but over time it drives maladaptive remodelling and represents a strong independent risk factor for HF [238]. Since hypertrophy in hypertension is, among others, related to cell death, fibrosis, and mitochondrial dysfunction [236], SIRT1 may have a potential therapeutic role, particularly in phenotypes characterized by dominant remodelling, with therapy potentially guided by CMR assessment of fibrosis burden. Therefore, perhaps males from cluster 2 would potentially benefit from such management. Additionally, patients with prominent metabolic syndrome (MetS)-related metabolic and inflammatory factors such as females from cluster 3 [234] and those with abnormal BMI more prone to insulin resistance could potentially benefit from SIRT1 level modulation. Experimental evidence supports this concept, as SIRT1 overexpression attenuated elevated BP and arterial remodeling through inhibition of the LKB1-TGFβ1 pathway, indicating a direct anti-remodeling role of SIRT1 in hypertension [239]. Given the central contribution of fibrosis and hypertrophy to heart failure progression, selective SIRT1 activation may be particularly attractive in hypertensive patients with early structural heart disease.

Coronary macro- and microvascular endothelial dysfunction can predict acute vascular events independently of conventional CAD risk factors and angiographically proven coronary atherosclerosis [10]. SIRT1 analogues could have potential applications across various forms of coronary artery disease that increase CV risk in hypertensive patients. SIRTs’ anti-inflammatory effects may be relevant in stable coronary artery disease with high-risk plaques identified on imaging. Ischemia with Non-Obstructive Coronary Artery disease involving the microvascular compartment, such as small intramural pre-arteriolar coronary arteries [10], also appears relevant in this context, particularly given that clinical outcomes with conventional treatment are often unsatisfactory. Profiling of micro- and macrovascular dysfunction in these patients is available using a panel of invasive measurements, including coronary vasomotor testing with intracoronary adenosine to evaluate coronary flow reserve, reflecting endothelium-independent microvascular function, acetylcholine to assess endothelium-dependent coronary vasoreactivity, and nitroglycerin to measure endothelium-independent macrovascular function [10]. Beyond functional vasomotion, SIRT1 may also influence plaque biology. Endothelial SIRT1 overexpression reduced atherosclerosis in ApoE-deficient mice, inhibited oxLDL-induced apoptosis, enhanced eNOS signaling, and improved endothelial function, suggesting that SIRT1 activation may stabilize plaques and attenuate atherogenesis [240]. This mechanism could be especially relevant in hypertensive patients with concomitant coronary atherosclerosis.

Arterial stiffness, commonly assessed by pulse wave velocity (PWV), is an independent predictor of coronary artery disease and stroke and is strongly associated with hypertension [241]. Longitudinal data indicate that PWV rises more steeply with age in men than in women [242]. Since SIRT1 signaling is linked to vascular smooth muscle phenotype, endothelial integrity, oxidative stress suppression, and extracellular matrix regulation, reduced SIRT1 activity may contribute to progressive arterial stiffening. Supporting this concept, activation of SIRT1 with SRT1720 attenuated Klotho deficiency-induced arterial stiffness and hypertension through enhancement of AMPK signaling [243]. Thus, SIRT1-targeted therapy may be particularly relevant in older patients with isolated systolic hypertension and increased pulse pressure.

Apart from hypertension, the role of SIRTs was discussed in atherosclerosis, aortic aneurysm/aortic dissection, diabetes-related vascular dysfunction, arterial calcification, cardiac hypertrophy and fibrosis, cardiotoxicity, cardiac ischemia–reperfusion injury and HF [244]. Other potential cardiology conditions in the context of SIRTs, with a plausible rationale for investigation, include mitral annulus disjunction, degenerative valvular heart disease, constrictive pericarditis, and arrhythmia such as atrial fibrillation. Moreover, hypertension frequently coexists with metabolic syndrome, characterized by abdominal obesity, hypertriglyceridemia, low HDL cholesterol, and impaired glucose metabolism. SIRT1 modulates each of these components through anti-inflammatory, anti-atherosclerotic, vasodilatory, and insulin-sensitizing effects. Therefore, restoration of SIRT1 activity may offer a multidimensional therapeutic strategy simultaneously targeting BP, vascular remodeling, and cardiometabolic risk.

**Table 6:** SIRT1 abundance and levels in human observational studies of hypertension.

Type of Hypertension	Participants Characteristics	Participants Amount	SIRT1 Level in Serum	Comments	Ref.
primary hypertension	age 8–17 years, males and females	67 males, 20 females	↓	(1) there was a significant negative correlation between SIRT1 level and BP in males, but not in females (2) SIRT1 level was lower in LVH group (3) SIRT1 was negatively correlated with LV mass Z-score	[245]
primary hypertension	age 15–80 years	40 hypertensive and 26 diabetic + hypertensive	↓ in DM + HTN group	(1) SIRT1 level was lower than in the pre-hypertensive group in both groups (2) SIRT1 was positively correlated with MAP at the normal range, but negatively with higher MAP (3) SIRT1 was positively correlated with Klotho level	[246]
pregnancy-induced hypertension	healthy pregnant women at a gestational age of 24–36 weeks	30	↓	-	[183]
pregnancy-induced hypertension	pregnant women	32	↔	-	[220]
preeclampsia	pregnant women	76	↓	(1) ↓ SIRT1 in placentas from 7 woman with pre-eclampsia with severe features	[221]
preeclampsia	pregnant women	31	↓	-	[215]
preeclampsia	pregnant women	62 with mild PE + 38 with severe PE	in severe group ↓ than in mild	(1) SIRT1 level was negatively correlated with SBP, DBP and random urinary protein level (2) SIRT1 has a high diagnostic value for the severity of PE	[214]
preeclampsia	pregnant women, severe PE	40	↓	(1) ↓ SIRT1 level also in placentas (2) positive correlation of SIRT1 level with serum PIGF level (3) negative correlation of SIRT1 level with serum sFlt-1 level	[216]
preeclampsia	pregnant women, severe PE	20	↓ in placentas	-	[187]
preeclampsia	pregnant women, premature placental aging and grade III placenta prior to 37 weeks gestation	10	↓ in placentas	-	[217]
preeclampsia	pregnant women	76	↓ in plasma	(1) SIRT1 level was lower in PE group than in GH group	[218]
gestational hypertension	pregnant women	70	↔ in plasma		

Abbreviations: BP, blood pressure; DM, diabetes mellitus; GH, gestational hypertension; HTN, hypertension; LV, left ventricle; LVH, left ventricular hypertrophy; MAP, mean arterial pressure; PE, pre-eclampsia; PIGF, placental growth factor; sFlt-1, soluble FMS-like tyrosine kinase-1; SaβG, senescence-associated β-galactosidase.

**Table 7:** Clinical trials of SIRT1 activation in hypertension.

NCT Number, Ref.	Title	Type	Phase	Status	Enrollment	Participants Characteristics	Interventions	Outcomes	
								BP	Changes in Other Parameters
NCT02616822 [222]	Acute Effects Of Trans-Resveratrol in Endothelial Function in Treated Hypertensive Patients	randomized, crossover, double-blind	1	completed	24 (10 M, 14 F)	age: 45–65 with treated HTN, with endothelial dysfunction	trans-resveratrol or placebo, 300 mg, single dose	↔	↑ FMD
NCT06828211	Prospective Open Randomized Controlled Trial on the Effectiveness and Tolerability of Trans-resveratrol (derived from Polygonum Cuspidatum Root Extr.) on the Development of CHF in Postmenopausal Women with HTN and Reduced BMD	randomized, parallel, open label	n/a	active, not recruiting	80	age: 45–55 female postmenopausal (1–4 years) with HTN (stage I-II) and decreased bone mineral density	trans-resveratrol or placebo, 500 mg daily, 3 months	ongoing	
NCT01691404 [213,223,247]	Study on the Effects of Epicatechin and Quercetin Supplementation on Vascular Function and Blood Pressure (FLAVO) in Untreated (Pre)Hypertensive Subjects	randomized, crossover, triple-blind	n/a	completed	38 (37 *)	age: 30–80 with primary HTN/ prehypertensive	quercetin-3-glucoside or placebo, 160 mg daily, 20 weeks	↔	↓ IL-1β
NCT04790942	Effect of Berberine Hydrochloride on Blood Pressure and Vascular Endothelial Function in Patients With Hypertension	randomized, parallel, double-blind	4	unknown	40	age: 18–75 with primary HTN	berberine hydrochloride tablets, 0.4g 3 times/day, 3 months or healthy lifestyle	unknown	

Abbreviations: F, female; HTN, hypertension; M, male; NCT, National Clinical Trial; FMD, flow-mediated dilation; * enrollment differs between references.

## 5. Limitations

Despite growing evidence supporting the involvement of SIRT1 in the pathophysiology of hypertension and related CV complications, several important limitations should be acknowledged. First, in many experimental and clinical studies it remains unclear whether the observed effects are mediated entirely or only partially through SIRT1 signaling. This issue is particularly relevant for studies employing activators such as REV, which is known to exert pleiotropic and non-specific effects, potentially impeding the interpretation of SIRT1-dependent mechanisms [58,73].

A major limitation is the inconsistent use of SIRT1 inhibition strategies across the included studies. While some authors confirmed the role of SIRT1 using specific inhibitors [136,175], the majority of studies did not employ such approaches. As a result, it cannot be definitively determined whether the observed effects are exclusively mediated by SIRT1 or involve additional signaling pathways. The lack of consistent mechanistic validation reduces the strength of causal inference and highlights the need for more standardized experimental designs incorporating both activation and inhibition of SIRT1.

The lack of precise data on BP values is another important limitation of the available studies. In several cases, only graphs were provided, or the degree of change was described without exact values. Additionally, different studies report distinct BP parameters, such as SBP, DBP and MAP, often without including all of them simultaneously . This heterogeneity in outcomes limits direct comparisons across studies and complicates the interpretation of the overall effect of SIRT1 activation on BP. Therefore, future studies should aim to report complete and standardized BP parameters to improve comparability and reproducibility of findings.

A significant limitation of several clinical investigations is their small sample size and single-centre design [see Table 6 and Table 7]. Studies conducted in pediatric or pregnancy-associated hypertension populations often included a limited number of participants, frequently with underrepresentation of female subjects, which reduced statistical power and limited the generalizability of their findings. Moreover, the lack of long-term follow-up in these studies precluded assessment of whether alterations in SIRT1 levels influence the development of primary hypertension and LV hypertrophy complications [245].

Furthermore, clinical translation of SIRT1 activators remains complex and partially inconsistent. In two cited clinical trials, administration of natural SIRT1 modulators failed to demonstrate reductions in systemic BP; nevertheless, they exerted distinct vascular and anti-inflammatory benefits, as evidenced by improved FMD following trans-resveratrol supplementation and reduced IL-1β levels with quercetin treatment [NCT02616822, NCT01691404]. These discrepancies between BP lowering and CV markers highlight potential limitations regarding bioavailability, treatment duration, dosage optimization, and cohort heterogeneity in human studies compared to preclinical models.

In addition, some clinical studies relied solely on observational data without validation in animal models, which limits causal inference and does not establish whether altered SIRT1 signaling precedes or follows hypertension. Conversely, there were experimental studies that did not include complementary in vivo experiments to confirm their proposed signaling pathways under conditions of genetic or pharmacological SIRT1 inhibition [212]. The absence of experiments assessing whether observed molecular or phenotypic effects were abolished in SIRT1-deficient models weakens causal interpretation and reduces the strength of the conclusions.

Methodological constraints also represent an important limitation. In certain studies, biomarker measurements were performed only once per sample due to financial constraints, which may increase susceptibility to analytical variability despite the use of standardized assays and certified clinical laboratories [246]. Furthermore, some experimental approaches lacked appropriate control conditions, such as inhibitor-only treatments, making it difficult to distinguish additive, synergistic, or antagonistic effects of pharmacological agents targeting SIRT1-related pathways [181].

Additional limitations arise from potential confounding factors related to biological heterogeneity. The effects of SIRT1 modulation may differ between sexes and age groups due to variations in hormonal regulation, CV physiology, and tissue-specific expression of related transporters and enzymes [181]. Moreover, in studies investigating nutraceuticals or plant-derived compounds, the presence of unidentified micronutrients may have contributed to off-target effects, complicating the interpretation of anti-apoptotic or anti-inflammatory outcomes. Technical limitations of commonly used assays, such as the inability of TUNEL staining to differentiate apoptosis from necrosis, further restrict definitive conclusions regarding cell death mechanisms [149].

Finally, several studies demonstrated beneficial CV or neuroprotective effects of specific interventions without fully elucidating the underlying molecular mechanisms. Insufficient investigation of central nervous system cell populations, such as glial cells or microglia, represents a gap in understanding the neurogenic contribution to hypertension and cardiac remodeling [174].

Taken together, these limitations highlight the need for well-powered, multi-centre, prospective clinical studies combined with robust mechanistic validation in animal and cellular models. Future research should employ selective genetic and pharmacological tools to clearly delineate SIRT1-dependent effects, incorporate long-term follow-up, and consider sex- and age-related differences to strengthen translational relevance.

## 6. Conclusions

Hypertension is a multifactorial disease driven by complex interactions between metabolic, inflammatory, neurohormonal and vascular mechanisms. The evidence summarized in this review suggests that SIRT1 participates in several processes relevant to hypertension through its ability to preserve endothelial function, maintain redox balance, improve mitochondrial homeostasis, attenuate inflammation and fibrosis, and delay cellular senescence. Experimental studies have shown favorable effects of SIRT1 modulation on vascular function and hypertension-mediated organ damage in several models; however, effects on blood pressure are less consistent and may depend on the experimental model and intervention used.

Although findings from animal models are promising, clinical evidence remains limited. Available human studies report associated altered SIRT1 expression or activity and hypertension or preeclampsia, but cannot establish whether these changes are a cause, consequence or compensatory response to elevated blood pressure. Early investigations of compounds proposed to activate SIRT1 suggest favorable effects on endothelial function and inflammatory status, although their effects cannot always be attributed specifically to SIRT1. These observations require confirmation in adequately powered, well-designed clinical trials evaluating long-term CV outcomes.

The potential value of SIRT1 as a biomarker of vascular aging, endothelial dysfunction and metabolic disturbances remains to be established. A better understanding of SIRT1-dependent signaling networks, interactions with NAD^+^ metabolism, mitochondrial function and currently available cardiometabolic therapies may facilitate the development of more targeted therapeutic approaches. Future translational and clinical studies should distinguish SIRT1 expression from enzymatic activity and determine whether SIRT1 modulation improves clinically relevant outcomes, including blood pressure control and the prevention of hypertension-mediated organ damage.

## Data Availability

No new data were created or analyzed in this study. Data sharing is not applicable to this article.

## Supplementary Materials

The Supplementary Material details the literature search process for evaluating SIRT1 role in hypertension. It provides a complete breakdown of search term groups and describes inclusion and exclusion criteria. The following supporting information can be downloaded at: https://www.mdpi.com/article/10.3390/antiox15101353/s1, Table S1: Search strategy and selection criteria.

## Abbreviations

The following abbreviations are used in this manuscript:

2′-*O*-AADPR	2′-*O*-acetyl-ADP-ribose
3-NT	3-nitrotyrosine
4HNE	4-hydroxy-2-nonenal
8-OHdG	8-hydroxy-2′-deoxyguanosine
ACE1	angiotensin converting enzyme 1
Ach	acetylcholine
ACR	albumin to creatinine ratio
ACTH	adrenocorticotropic hormone
AIF	alpinumisoflavone
Akt	protein kinase B
ALT	alanine transaminase
AMPK	adenosine 5′-monophosphate-activated protein kinase
Ang II	angiotensin II
ANP	atrial natriuretic peptide
Apaf-1	apoptotic protease activating factor-1
APP	amyloid precursor protein
ARE	antioxidant response elements
AROS	active regulator of SIRT1
ASC	apoptosis-associated speck-like protein containing a caspase recruitment domain
AST	aspartate aminotransferase
AT1-AA	autoantibodies against angiotensin II type 1 receptor
AT1R	angiotensin II type 1 receptor
ATF6	activating transcription factor 6
ATP	adenosine triphosphate
AVP	arginine vasopressin
Bad	Bcl-2-associated death promoter
BAIBA	β-aminoisobutyric acid
Bax	Bcl-2-associated X protein
Bcl-2	B-cell lymphoma-2
Bcl-xl	B-cell lymphoma-extra large
BMI	body mass index
BMTN	bumetanide
Bnip3	*Bcl-2 interacting protein 3*
BNP	brain natriuretic peptide
BS/GS	Bartter’s or Gitelman’s syndromes
BUN	blood urea nitrogen
BW	body weight
cAMP	3′,5′-cyclic adenosine monophosphate
CCND1	cyclin D1
Cdk4	cyclin-dependent kinase 4
cGMP	cyclic guanosine monophosphate
CHOP	C/EBP homologous protein
CK	creatinine kinase
CMR	cardiac magnetic resonance
CO	cardiac output
COL1A1	collagen type 1 α 1
COX-2	cyclo-oxygenase 2
CsA	cyclosporine A
CTGF	connective tissue growth factor
CTNNB1	catenin beta 1
CTR	C-terminal regulatory segment
CV	cardiovascular
CVD	cardiovascular diseases
DBP	diastolic blood pressure
DM	diabetes mellitus
Drp1	dynamin-related protein 1
ECs	endothelial cells
EF	ejection fraction
EGCG	epigallocatechin-3-gallate
EGF-R	epidermal growth factor receptor
empa	empagliflozin
EMT	epithelial–mesenchymal transition
EndMT	endothelial-to-mesenchymal transition
EndoG	endonuclease G
eNOS	endothelial nitric oxide synthase
EPAC1	cAMP dependent exchange protein 1
EPI	epinephrine
ERK1/2	extracellular signal-regulated kinase 1/2
ET-1	endothelin-1
ETC	electron transport chain
FADD	Fas-associated protein with death domain
Fas	type I integral membrane protein
Fas L	Fas ligand
FGF21	fibroblast growth factor
FMD	flow-mediated dilation
FN	fibronectin
FOXO1	forkhead box protein O1
FOXO3	forkhead box O3
FS	fractional shortening
FTH1	ferritin heavy chain 1
GAD67	67 kDa glutamic acid decarboxylase
Gclc	glutamate-cystine ligase catalytic subunit
Gclm	cystine ligase modifier subunit
GD	gestational day
GDF-15	growth differentiation factor 15
Gen	geniposide
GH	gestational hypertension
Giα-2	inhibitory guanine nucleotide regulatory protein α-2
gp91^phox^	glycoprotein of 91 kDa phagocyte oxidase
GPX4	glutathione peroxidase 4
GRP78	glucose related protein 78
GSH	glutathione
GSH-Px	glutathione peroxidase
GSSG	glutathione disulfide
H3K27ac	histone 3 lysine 27 acetylation
H3K9ac	histone H3 lysine 9 acetylation
HDAC1	histone deacetylase 1
HDL	high-density lipoprotein
HF	heart failure
HFD	high-fat diet
HIF-1	hypoxia-inducible factor-1
HMOD	hypertension-mediated organ damage
HO-1	heme oxygenase-1
HPVECs	human placental vascular endothelial cells
HR	heart rate
HS	high salt
HSP90AA1	heat shock protein 90 alpha family class A member 1
HSP90AB1	heat shock protein 90 alpha family class B member 1
HTN	hypertension
HUVECs	human umbilical venous endothelial cells
IBMX	3-isobutyl-1-methylxanthine
ICAM-1	intercellular adhesion molecule 1
ICV	intracerebroventricular
IDH	isolated diastolic hypertension
IF	Isoleucine-Phenylalanine dipeptide
IGF1	insulin-like growth factor 1
IGF-II	insulin-like growth factor-II
IL-1β	interleukin-1β
IL-6	interleukin-6
iNOS	inducible nitric oxide synthase
ISH	isolated systolic hypertension
IVSd	interventricular septum at diastole
IκBα	inhibitor of NF-κB
IΚΚβ	IκB kinase beta
JNK	c-Jun N-terminal kinase
K.pn. EVs	Klebsiella pneumoniae-secreted extracellular vesicles
k_cat_	catalytic constant
KCl	potassium chloride
Keap1	Kelch-like ECH-associated protein 1
K_m_	Michaelis constant
LC3-II	microtubule-associated protein 1A/1B-light chain 3-phosphatidylethanolamine conjugate
LDH	lactate dehydrogenase
LDL-C	low-density lipoprotein cholesterol
L-NAME	L-N^G^-Nitro arginine methyl ester
LVH	left ventricular hypertrophy
MACE	major adverse cardiovascular event
MAPK	mitogen-activated protein kinase
MAs	mesenteric arteries
MCP-1	monocyte chemoattractant protein 1
MetS	metabolic syndrome
Mfn1/2	mitofusin 1 and 2
MMPs	matrix metalloproteinases
MnSOD	manganese superoxide dismutase
MOI	multiplicity of infection
mtDNA	mitochondrial DNA
mTOR	mechanistic target of rapamycin
MV E/A	mitral valve early diastolic to late diastolic wave velocity ratio
MYH7	myosin heavy chain 7
NAD^+^	nicotinamide adenine dinucleotide
NADPH	reduced nicotinamide adenine dinucleotide phosphate
NAM	nicotinamide
NE	norepinephrine
NFATC3	nuclear factor of activated T cells 3
NF-κB	nuclear factor kappa-light-chain-enhancer of activated B cells
NLRP3	pyrin domain-containing protein 3
NO	nitric oxide
NOX	NADPH oxidase
NPPA	natriuretic peptide precursor A
NPPB	natriuretic peptide precursor B
Nqo1	NAD(P)H:quinone oxidoreductase
Nrf1	nuclear respiratory factor 1
Nrf2	nuclear factor erythroid 2-related factor 2
n-tyr	tyrosine nitration
OE	overexpression
ONOO^−^	peroxynitrite
Opn	osteopontin
p21	cyclin-dependent kinase inhibitor 1A
p300	E1A-associated 300 kDa protein
p47^phox^	cytosolic subunit of the NADPH oxidase
P53	tumor protein 53
p55	palmitoylated erythrocyte membrane protein
p65	activated B cells protein
Pae	paeoniflorin
PAMK	polysaccharide of Atractylodes macrocephala Koidz
PCNA	proliferating cell nuclear antigen
PE	preeclampsia
PERK	protein kinase RNA-activated-like endoplasmic reticulum kinase
PGC-1α	peroxisome proliferator-activated receptor-γ coactivator 1-α
PGI_2_	prostaglandin I2
PGQYD	Pinggan-Qianyang decoction
Phe	phenylephrine
PI3K	phosphoinositide 3-kinase
PIK3CA	phosphatidylinositol-4,5-bisphosphate 3-kinase catalytic subunit alpha
PKA	protein kinase A
PlGF	placental growth factor
PP	pulse pressure
PPARG	peroxisome proliferator-activated receptor gamma
PPARα	peroxisome proliferator-activated receptor α
PRKCA	protein kinase C alpha
PTEN	phosphatase and tensin homolog
PTGS2	prostaglandin-endoperoxide synthase
PVAT	perivascular adipose tissue
PVN	paraventricular nucleus
PVR	peripheral vascular resistance
QYYYG	Qianyang Yuyin granule
RAA	renin–angiotensin–aldosterone
Rb	retinoblastoma protein
REV	resveratrol
ROS	reactive oxygen species
RSNA	renal sympathetic nerve activity
rSHH	recombinant Sonic Hedgehog protein
RVLM	rostral ventrolateral medulla
SA-β-gal	senescence-associated β-galactosidase
SBP	systolic blood pressure
SCR	serum creatinine
SD	Sprague Dawley
SDH	systolic–diastolic hypertension
sFlt-1	soluble FMS-like tyrosine kinase-1
SGLT2	sodium-glucose cotransporter 2
SHR	spontaneously hypertensive rat
shRNA	short hairpin RNA
si-PDE4B	phosphodiesterase 4B small interfering RNA
SIRT1	sirtuin 1
SIRT2	sirtuin 2
SIRT3	sirtuin 3
siSIRT1	small interfering SIRT1 RNA
SM22	smooth muscle 22α
Smad	mothers against decapentaplegic homolog
SNS	sympathetic nervous system
SOD	superoxide dismutase
sPE	severe preeclampsia
STAT3	signal transducer and activator of transcription 3
SV	stroke volume
T-AOC	total antioxidant capacity
t-Bid	truncated BH3-interacting domain death agonist
T-CHO	total cholesterol
TFAM	mitochondrial transcription factor A
TGFβ	transforming growth factor β
TLR4	tool-like receptor 4
TNFα	tumor necrosis factor-α
TP	TxA_2_/prostanoid
TXA_2_	thromboxane A2
uPA	urokinase-type plasminogen activator protein
V1R	vasopressin type 1a receptor
VCAM-1	vascular cell adhesion molecule
VEGF	vascular endothelial growth factor
VH4	Valine–Histidine–Valine–Valine peptide
VSMCs	vascular smooth muscle cells
WoS	Web of Science
WT	weight
α-MG	alpha-mangostin
α-SMA	alpha smooth muscle actin
