# Supplementary material for: Sirtuin-1 in Hypertension: Linking Oxidative Stress and Mitochondrial Dysfunction to Vascular Aging and Therapeutic Potential
Source: Antioxidants (Basel). 2026 Oct 20;15(10):1353. doi: 10.3390/antiox15101353 (PMC13701266; doi:10.3390/antiox15101353)
Supplement: Supplementary file 1 [file antioxidants-15-01353-s001.zip › antioxidants-4513603-supplementary.pdf]

Supplementary Material S1.

This review was conducted to evaluate SIRT1 in hypertension using predefined search keywords across various domains, including human studies and clinical trials. Identified records were screened, with all duplicates, preprints, and retractions systematically quantified.

The inclusion criteria required publications to be original research articles published in English that investigated SIRT1 in the context of hypertension or related cardiovascular mechanisms and provided relevant experimental or clinical data.

Records were excluded if they were non-original papers, non-English publications, lacked full-text access, or were irrelevant. The exclusion criterion "not relevant" defines publications that either focused exclusively on diseases unrelated to hypertension or failed to provide relevant information regarding SIRT1 expression, activity, regulation, or functional effects in hypertension. Furthermore, within the human studies sections, the specific exclusion phrase "not relevant to this review or this section" denotes that the rejected publications diseases unrelated to hypertension or failed to provide relevant information regarding SIRT1 role in hypertension or were not based on human subjects.

Regarding clinical trials evaluating hypertension in conjunction with specific interventions, records were specifically excluded if the trials were terminated or deemed not suitable for the review based on the provided treatment description.

Table S1. Search strategy and selection criteria.

Search Term Group	Search Keywords	Records Identified (Duplicates / Preprints / Retracted)	Exclusion Criteria	Excluded Records (n)	No Full Text (n)	Included Records (n)
1	SIRT1 OR silent information regulator 1 AND systemic hypertension	169 (0/0/3)	not original papers	42	1	34
			not in English	3		
			not about SIRT1 and hypertension (based on title)	74		
			not about SIRT1 and hypertension (based on abstracts and full texts)	1		
			about SIRT1 but not relevant to this review (based on title)	9		
			about SIRT1 but not relevant to this review (based on abstracts and full texts	2		
2	SIRT1 OR silent information regulator 1 AND blood pressure	116 (43/0/0)	not original papers	4	0	2
			not in English	1		
			not about SIRT1 and hypertension (based on title)	59		
			not about SIRT1 and hypertension (based on abstracts and full texts)	5		

			about SIRT1 but not relevant to this review (based on title)	0		
			about SIRT1 but not relevant to this review (based on abstracts and full texts	2		
3	SIRT1 OR silent information regulator 1 AND elevated blood pressure	180 (175/0/0)	not original papers	0	0	0
			not in English	0		
			not about SIRT1 and hypertension (based on title)	5		
			not about SIRT1 and hypertension (based on abstracts and full texts)	0		
			about SIRT1 but not relevant to this review (based on title)	0		
			about SIRT1 but not relevant to this review (based on abstracts and full texts	0		
4	SIRT1 OR silent information regulator 1 AND vascular hypertension	202 (202/0/0)	not original papers	0	0	0
			not in English	0		
			not about SIRT1 and hypertension (based on title)	0		
			not about SIRT1 and hypertension (based on abstracts and full texts)	0		

			about SIRT1 but not relevant to this review (based on title)	0		
			about SIRT1 but not relevant to this review (based on abstracts and full texts	0		
5	SIRT1 OR silent information regulator 1 AND endothelial dysfunction	192 (39/1/1)	not original papers	21	0	0
			not in English	1		
			not about SIRT1 and hypertension (based on title)	120		
			not about SIRT1 and hypertension (based on abstracts and full texts)	9		
			about SIRT1 but not relevant to this review (based on title)	0		
			about SIRT1 but not relevant to this review (based on abstracts and full texts	0		
6	SIRT1 OR silent information regulator 1 AND vascular remodeling	50 (33/0/0)	not original papers	5	0	0
			not in English	1		
			not about SIRT1 and hypertension (based on title)	11		
			not about SIRT1 and hypertension (based on abstracts and full texts)	0		

			about SIRT1 but not relevant to this review (based on title)	0		
			about SIRT1 but not relevant to this review (based on abstracts and full texts	0		
7	SIRT1 OR silent information regulator 1 AND vascular ageing	120 (65/0/0)	not original papers	11	0	0
			not in English	3		
			not about SIRT1 and hypertension (based on title)	33		
			not about SIRT1 and hypertension (based on abstracts and full texts)	6		
			about SIRT1 but not relevant to this review (based on title)	0		
			about SIRT1 but not relevant to this review (based on abstracts and full texts	2		
8	SIRT1 OR silent information regulator 1 AND cellular senescence	281 (53/3/0)	not original papers	29	0	1
			not in English	1		
			not about SIRT1 and hypertension (based on title)	178		
			not about SIRT1 and hypertension (based on abstracts and full texts)	15		

			about SIRT1 but not relevant to this review (based on title)	0		
			about SIRT1 but not relevant to this review (based on abstracts and full texts	1		
9	SIRT1 OR silent information regulator 1 AND mitochondrial dysfunction	943 (105/2/2)	not original papers	167	0	1
			not in English	9		
			not about SIRT1 and hypertension (based on title)	655		
			not about SIRT1 and hypertension (based on abstracts and full texts)	2		
			about SIRT1 but not relevant to this review (based on title)	0		
			about SIRT1 but not relevant to this review (based on abstracts and full texts	0		
10	SIRT1 OR silent information regulator 1 AND oxidative stress	2301 (686/1/11)	not original papers	218	0	0
			not in English	14		
			not about SIRT1 and hypertension (based on title)	1363		
			not about SIRT1 and hypertension (based on abstracts and full texts)	8		

			about SIRT1 but not relevant to this review (based on title)	0		
			about SIRT1 but not relevant to this review (based on abstracts and full texts	0		
11	SIRT1 OR silent information regulator 1 AND inflammation	1983 (1141/2/1)	not original papers	98	0	0
			not in English	9		
			not about SIRT1 and hypertension (based on title)	732		
			not about SIRT1 and hypertension (based on abstracts and full texts)	0		
			about SIRT1 but not relevant to this review (based on title)	0		
			about SIRT1 but not relevant to this review (based on abstracts and full texts	0		
**human studies**						
12	SIRT1 AND systemic hypertension AND patients	72 (0/0/0)	not original papers	16	0	5
			not in English	1		
			not about SIRT1 and hypertension (based on title)	43		

			not about SIRT1 and hypertension (based on abstracts and full texts)	1		
			about SIRT1 but not relevant to this review or this section (based on title)	4		
			about SIRT1 but not relevant to this review or this section (based on abstracts and full texts	2		
13	SIRT1 AND systemic hypertension AND humans	202 (55/0/0)	not original papers	61	0	2
			not in English	1		
			not about SIRT1 and hypertension (based on title)	72		
			not about SIRT1 and hypertension (based on abstracts and full texts)	0		
			about SIRT1 but not relevant to this review or this section (based on title)	5		
			about SIRT1 but not relevant to this review or this section (based on abstracts and full texts	6		
14		44 (7/0/1)	not original papers	4	0	6

	SIRT1 AND preeclampsia		not in English	0		
			not about SIRT1 and hypertension (based on title)	17		
			not about SIRT1 and hypertension (based on abstracts and full texts)	0		
			about SIRT1 but not relevant to this review or this section (based on title)	7		
			about SIRT1 but not relevant to this review or this section (based on abstracts and full texts	2		
duplicates between human and animal studies						4
**clinical trials**						
15	Hypertension AND Resveratrol	5	Trial terminated	1	0	2
			Not suitable for this review (based on treatment description)	2		
16	Hypertension AND Amifostine	0	Trial terminated	0	0	0
			Not suitable for this review (based on treatment description)	0		
17	Hypertension AND Quercetin	3	Trial terminated	1	0	1
			Not suitable for this review (based on treatment description)	1		

18	Hypertension AND Berberine	4	Trial terminated	0	0	1
			Not suitable for this review (based on treatment description)	3		
19	Hypertension AND NAMPT	0	Trial terminated	0	0	0
			Not suitable for this review (based on treatment description)	0		
20	Hypertension AND Fisetin	0	Trial terminated	0	0	0
			Not suitable for this review (based on treatment description)	0		
21	Hypertension AND AMPK	1	Trial terminated	0	0	0
			Not suitable for this review (based on treatment description)	1		
22	Hypertension AND Curcumin	5	Trial terminated	2	0	0
			Not suitable for this review (based on treatment description)	3		
23	Hypertension AND Ferulic acid	0	Trial terminated	0	0	0
			Not suitable for this review (based on treatment description)	0		
24	Hypertension AND SRT1720	0	Trial terminated	0	0	0
			Not suitable for this review (based on treatment description)	0		
25		0	Trial terminated	0	0	0

	Hypertension AND SRT2104		Not suitable for this review (based on treatment description)	0		
26	Hypertension AND Stac-3	0	Trial terminated	0	0	0
			Not suitable for this review (based on treatment description)	0		
27	Hypertension AND Jagged 2 inhibitor	0	Trial terminated	0	0	0
			Not suitable for this review (based on treatment description)	0		
28	Hypertension AND 15-HETE	0	Trial terminated	0	0	0
			Not suitable for this review (based on treatment description)	0		
29	Hypertension AND VHVV	0	Trial terminated	0	0	0
			Not suitable for this review (based on treatment description)	0		
30	Hypertension AND IF	0	Trial terminated	0	0	0
			Not suitable for this review (based on treatment description)	0		
31	Hypertension AND Klotho	3	Trial terminated	0	0	0
			Not suitable for this review (based on treatment description)	3		
